# Ruthenium(II) Complex with 1-Hydroxy-9,10-Anthraquinone Inhibits Cell Cycle Progression at G0/G1 and Induces Apoptosis in Melanoma Cells

**DOI:** 10.3390/ph18010063

**Published:** 2025-01-08

**Authors:** Júlia S. M. Dias, Guilherme A. Ferreira-Silva, Rommel B. Viana, João H. de Araujo Neto, Javier Ellena, Rodrigo S. Corrêa, Marília I. F. Barbosa, Marisa Ionta, Antônio C. Doriguetto

**Affiliations:** 1 Instituto de Química, Universidade Federal de Alfenas (UNIFAL-MG), Alfenas 37130-000, MG, Brazil; scaffjulia@gmail.com (J.S.M.D.); mariliaifrazaob@gmail.com (M.I.F.B.); 2 Departamento de Ciências Biomédicas, Universidade Federal de Alfenas (UNIFAL-MG), Alfenas 37130-000, MG, Brazil; alfer.guilherme@gmail.com; 3 Departamento de Química, Universidade Estadual do Ceará (UECE), Limoeiro do Norte 62930-000, CE, Brazil; rommel.viana@uece.br; 4 Instituto de Química, Universidade de São Paulo (USP), São Paulo 05508-000, SP, Brazil; joaohonorato@iq.usp.br; 5 Instituto de Física de São Carlos, Universidade de São Paulo (USP), São Carlos 13566-590, SP, Brazil; javiere@ifsc.usp.br; 6 Departamento de Química, Universidade Federal de Ouro Preto (UFOP), Ouro Preto 35400-000, MG, Brazil; rodrigocorrea@ufop.edu.br

**Keywords:** 1-hydroxy-9,10-anthraquinone, ruthenium complexes, melanoma, antiproliferative activity

## Abstract

Background: Melanoma is the most aggressive and lethal skin cancer that affects thousands of people worldwide. Ruthenium complexes have shown promising results as cancer chemotherapeutics, offering several advantages over platinum drugs, such as potent efficacy, low toxicity, and less drug resistance. Additionally, anthraquinone derivatives have broad therapeutic applications, including melanoma. Objectives: Thus, two new ruthenium complexes with 1-hydroxy-9,10-anthraquinone were obtained: *trans-*[Ru(HQ)(PPh_3_)_2_(bipy)]PF_6_ (**1**) and *cis-*[RuCl_2_(HQ)(dppb)] (**2**), where HQ = 1-hydroxy-9,10-anthraquinone, PPh_3_ = triphenylphospine, bipy = 2,2′-bipyridine, PF_6_ = hexafluorophosphate, and dppb = 1,4-bis(diphenylphosphine)butane. Methods: The complexes were characterized by infrared (IR), UV–vis, ^1^H, ^13^C{^1^H}, and ^31^P{^1^H} NMR spectroscopies, molar conductivity, cyclic voltammetry, and elemental analysis. Furthermore, density functional theory (DFT) calculations were performed. Results: Compound (**2**) was determined by single-crystal X-ray diffraction, which confirms the bidentate coordination mode of HQ through the carbonyl and phenolate oxygens. Additionally, DNA-binding experiments yielded constants of 10^5^ M^−1^ (Kb = 6.93 × 10^5^ for (**1**) and 1.60 × 10^5^ for (**2**)) and demonstrate that both complexes can interact with DNA through intercalation, electrostatic attraction, or hydrogen bonding. Conclusions: The cytotoxicity profiles of the compounds were evaluated in human melanoma cell lines (SK-MEL-147, CHL-1, and WM1366), revealing greater cytotoxic activity for (**1**) on the CHL-1 cell line with an IC_50_ of 14.50 ± 1.09 µM. Subsequent studies showed that (**1**) inhibits the proliferation of CHL-1 cells and induces apoptosis, associated at least in part with the pro-oxidant effect and cell cycle arrest at the G1/S transition.

## 1. Introduction

Melanoma is the most aggressive and lethal type of skin cancer, originating in melanocytes, which produce the pigment melanin that gives skin its color [1,2]. According to the International Agency for Research on Cancer (IARC), melanoma was responsible for approximately 330,000 new cancer cases worldwide and around 60,000 deaths in 2022 [3].

The incidence of melanoma is higher in the fair-skinned population [4], as the levels of melanin pigment, which protects against UV radiation, are low [5,6]. Additionally, melanoma metastasizes more easily than other types of skin cancer and can quickly become fatal [7]. Therefore, early diagnosis and the development of new therapies, which are the aim of this research, are extremely important.

**Figure 1:**
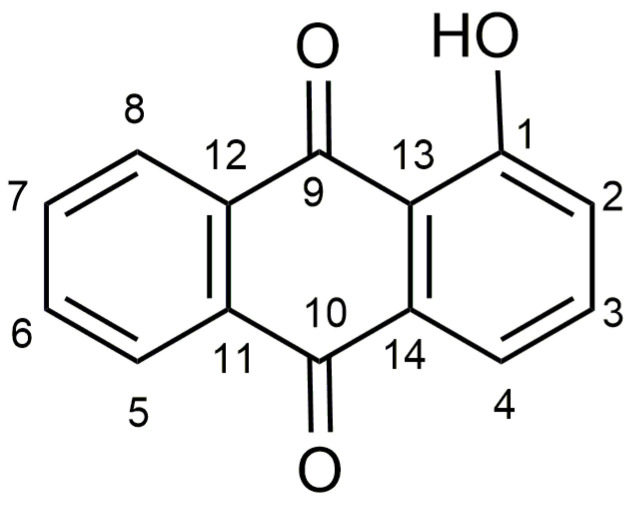
1-hydroxy-9,10-anthraquinone structure and ^13^C {^1^H} signal assignment.

Anthraquinone derivatives have broad therapeutic applications [8], particularly noted for their antitumor activity against various types of cancer [9,10,11], including melanoma [12,13,14,15]. The 1-hydroxy-9,10-anthraquinone HQ (Figure 1) is derived from the basic structure of 9,10-anthracenedione and includes a hydroxyl group, enabling it to coordinate with several metallic cations [16].

Over the past few decades, ruthenium complexes have shown promising results as cancer chemotherapeutics [17], offering several advantages over platinum drugs, such as potent efficacy, low toxicity, and less drug resistance. These characteristics position them as potential candidates for the next generation of clinical metal-based antitumor drugs [18,19,20]. Numerous studies have explored the combination of metallic cations and organic molecules with inherent biological activity to enhance their mechanisms of action [18].

The ruthenium complexes commonly adopt an octahedral geometry and present Ru(II) and Ru(III) as their main oxidation states [21]. Ru(III) complexes generally function as prodrugs, requiring reduction to Ru(II) under biological conditions, such as enzyme activity and a hypoxic environment [22,23]. The antitumor activity of Ru(II) complexes may involve various mechanisms of action and targets, including DNA, proteins, and cell organelles [24,25].

In this paper, we aimed to investigate the influence of HQ-Ru(II) and Ru(III) complexes on three melanoma cell lines (WM1366, CHL-1, and SK-MEL-147). Additionally, the interactions of the complexes with ct-DNA were evaluated and described. Furthermore, density functional theory (DFT) calculations were performed to investigate the electronic and thermodynamic properties of these complexes, and time-dependent density functional theory (TD-DFT) was employed to elucidate the intricate interplay of metal–ligand charge transfer processes in UV–vis electronic transitions.

## 2. Results and Discussion

### 2.1. Synthesis and Characterization of the Complexes

**Scheme 1:**
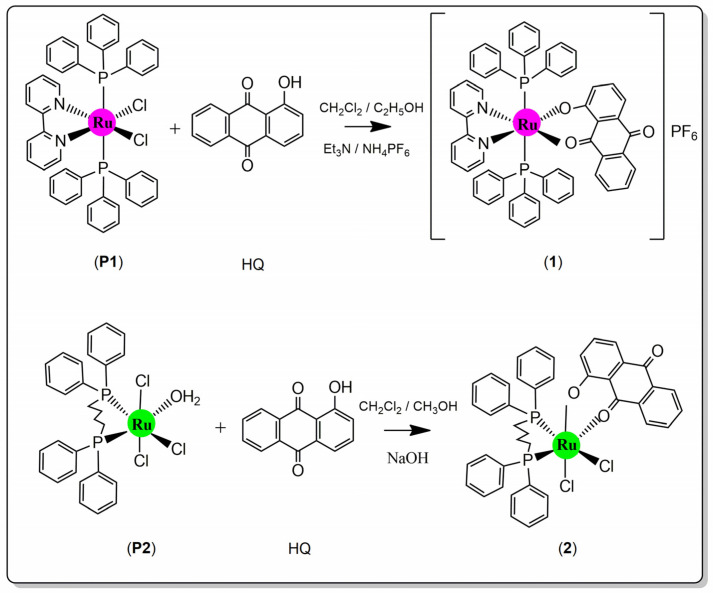
Synthetic route of complexes (**1**) and (**2**).

The reactions of HQ with ruthenium precursors *cis-*[RuCl_2_(PPh_3_)_2_(bipy)] (**P1**) [26] and *mer-*[RuCl_3_(dppb)(H_2_O)] (**P2**) [27] were employed to obtain the complexes *trans-*[Ru(HQ)(PPh_3_)_2_(bipy)]PF_6_ (**1**) and *cis*-[RuCl_2_(HQ)(dppb)] (**2**), where PPh_3_ = triphenylphospine, bipy = 2,2′-bipyridine, PF_6_ = hexafluorophosphate, and dppb = 1,4-bis(diphenylphosphine)butane. The synthesis occurred via the exchange of two chloride or chloride/water ligands under mild conditions (Scheme 1).

The elemental analyses, as described in the Section 3, were in agreement with the proposed formulations. The molar conductance values measured in dichloromethane at room temperature indicate a 1:1 electrolyte (46.50 S cm^2^ mol^−1^) for (**1**) and a neutral compound (0.34 S cm^2^ mol^−1^) for (**2**) [28], as expected.

Infrared spectra of the complexes were compared with the spectrum of the free ligand and displayed characteristic bands due to HQ and PPh_3_ for (**1**) and dppb for (**2**) (Supplementary Materials: Figures S1–S3). The spectrum of HQ shows bands at 1672 cm^−1^ and 1636 cm^−1^, corresponding to ν(C=O), and the band at 1290 cm^−1^ is characteristic of ν(C-O) (Table 1).

As expected, the ν(C=O) shifted to lower frequencies in the complexes (1649 and 1585 cm^−1^ for (**1**) and 1662 and 1611 cm^−1^ for (**2**)), indicating the weakening of the double bond after coordination to the metal, as reported for HQ metal complexes [29,30]. Conversely, the band related to ν(C-O) shifted to higher frequencies (1306 cm^−1^ for (**1**) and 1298 cm^−1^ for (**2**)), similar to molecules of analogous structure [31,32]. Thus, the displacement of these bands is in agreement with the coordination through the oxygen atoms of the carbonyl and phenolate groups. In addition, the spectrum of (**1**) shows two bands at 839 and 557 cm^−1^, assigned to the counter-ion PF_6_. The IR spectra of both complexes exhibited bands in the region from 518 to 496 cm^−1^, corresponding to the Ru-P stretching, while bands of weak intensities were assigned to ν(Ru–O) (492 cm^−1^ for (**1**) and 476 cm^−1^ for (**2**)) and 461 cm^−1^ for (**1**) ν(Ru–N) [33,34]. The bands corresponding to ν(C=O) showed smaller frequency variations for (**2**) compared to (**1**), demonstrating the influence of the Ru(III) metallic center and the phosphorous ligand *trans* to the carbonyl group of HQ. In contrast, compound (**1**) has the carbonyl group *trans* to the nitrogen atom and features a Ru(II) metallic center.

**Figure 2:**
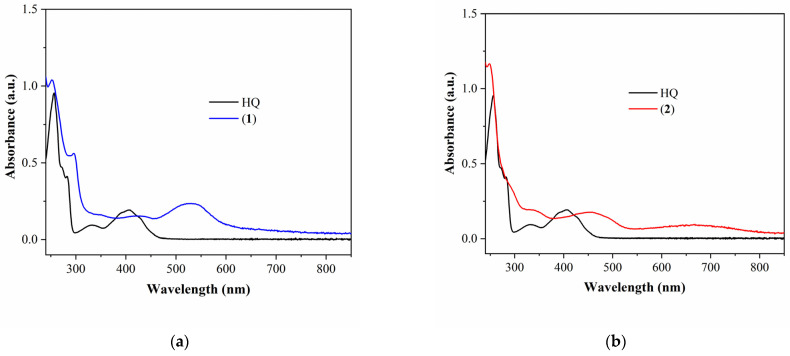
UV–vis absorption spectra in CH_2_Cl_2_ of (**a**) HQ 2.65 × 10^−5^ mol L^−1^ and (**1**) 1.27 × 10^−5^ mol L^−1^, and (**b**) HQ 2.65 × 10^−5^ mol L^−1^ and (**2**) 1.63 × 10^−5^ mol L^−1^.

The electronic spectrum of the ligand showed three bands at 257, 333, and 407 nm resulting from π-π* transitions and contributions from oxygen p-orbitals [35,36]. The UV–vis spectra of the complexes also showed three bands at 252, 296, and 528 nm for (**1**) (Figure 2a) and 248, 455, and 668 nm for (**2**) (Figure 2b).

The more intense bands at 252 nm and 296 nm for (**1**) have distinct contributions, including metal-to-ligand charge transfer (MLCT), metal-to-metal-to-ligand charge transfer (MMLCT), and metal-to-ligand-to-ligand charge transfer (MLLCT), as discussed in detail in Section 2.2 after a combined analysis of TD-DFT calculations and UV–vis analysis. The band at 528 nm can be attributed to the MLCT process from Ru(dπ) → HQ (π*), consistent with other studies involving Ru(II) complexes of similar structures [31,34,37], along with a minor contribution from MLMLCT (discussed in Section 2.2).

**Figure 3:**
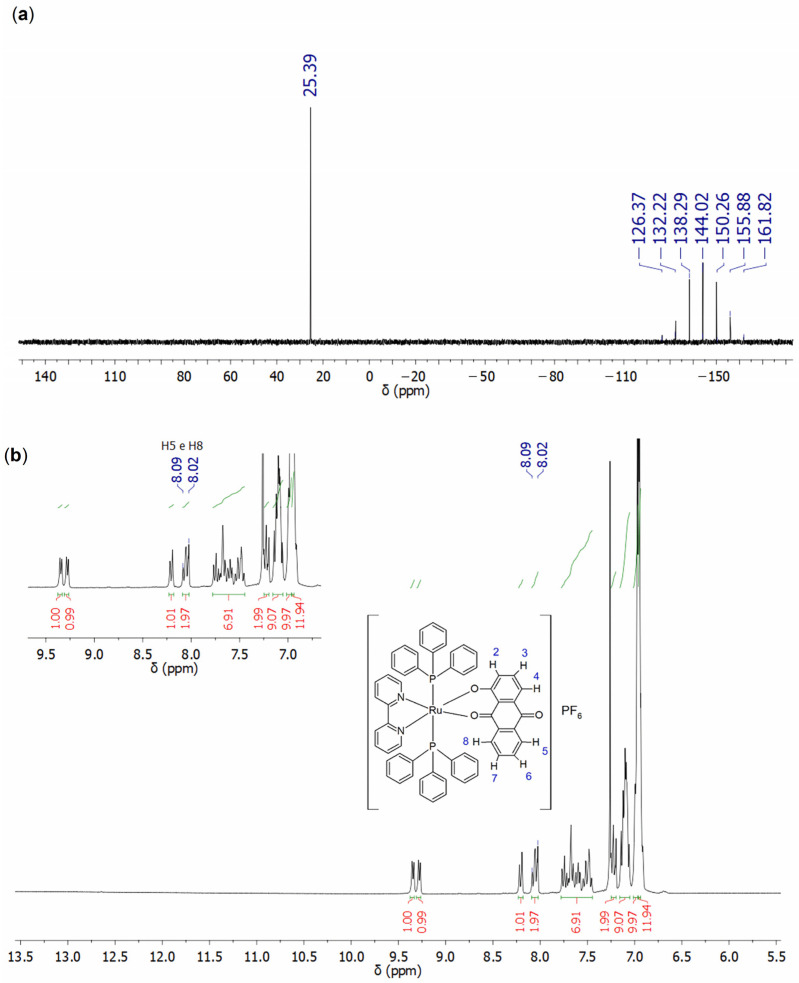
(**a**) ^31^P(^1^H) NMR spectrum for (**1**) in CDCl_3_ and (**b**) ^1^H NMR spectrum of (**1**) in CDCl_3_, and assignment of signals referring to the complex.

The broad band at 668 nm for (**2**) replicates the spectral profile recently reported by us [32] and in other works on Ru(III) complexes [38,39]. This band can be attributed to combined processes, namely MLLCT and MLMLCT. The other bands also exhibit diverse contributions, which will be described in detail Section 2.2. The ^31^P{^1^H} NMR spectrum of (**1**) in CDCl_3_ showed a singlet at 25.39 ppm (Figure 3a) indicating the magnetic equivalence of the two phosphorus atoms in a *trans-*configuration. Thus, (**1**) features a higher chemical shift than (**P1**), where the singlet signal occurs at 21.53 ppm [26]. The deshielding of the phosphorus nuclei is attributed to the exchange of two chlorine atoms (good electron donors) with oxygen atoms in HQ, which possess lower electron-donating character. This observation is consistent with reports for *trans-*[Ru(PPh_3_)_2_(O-O)(bipy)]PF_6_, where O-O = lawsone [31] or lapachol [34]. Furthermore, in the ^31^P{^1^H} NMR spectrum of (**1**), a septet is observed at −144 ppm, corresponding to the PF_6_ counter-ion.

In the ^1^H NMR spectrum of the free HQ, a singlet at 12.60 ppm was assigned to the hydroxyl group H1, and signals ranging from 8.33 to 7.29 ppm were assigned to H2–H8 (Supplementary Materials: Figure S4). For (**1**), the disappearance of the signal corresponding to H1 indicates the deprotonation of the OH group and its coordination with ruthenium (Figure 3b). Additionally, the chemical shifts of H5 and H8 showed characteristic shielded signals at 8.09 to 8.02 ppm compared to the free HQ ligand (8.33 to 8.28 ppm). Other aromatic hydrogen atom resonances were observed in the range of 7.77–6.95 ppm and were attributed to the protons present in the aromatic phosphine, HQ, and bipyridine ligands.

The ^13^C{^1^H} chemical shifts for the free HQ and for (**1**) are summarized in Table 2 and Figures S5 and S6 of the Supplementary Materials. Significant changes were observed for the signals corresponding to the carbons C1, C9, and C10. The signal corresponding to C1 shifted to higher frequencies from 162.90 ppm HQ to 170.56 ppm (**1**), indicating the deshielding of the nucleus, as well as the neighboring carbons C2 and C13. Additionally, C9 and C10 were strongly shielded (C9 shifted from 188.99 ppm HQ to 182.92 ppm and C10 shifted from 182.72 ppm HQ to 178.18 ppm for (**1**)), suggesting coordination by the carbonyl and phenolate groups. The NMR technique was not performed for (**2**) due to the paramagnetic nature of the Ru(III) metal center.

**Figure 4:**
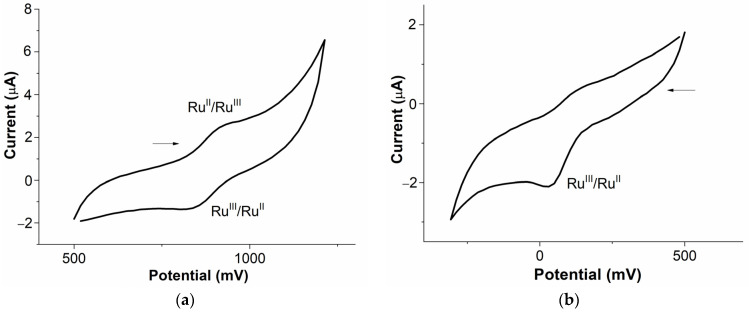
Cyclic voltammograms of complexes: (**a**) (**1**) and (**b**) (**2**) (TBAP 0.1 M; CH_2_Cl_2_; Ag/AgCl; work electrode Pt; 100 mV s^−1^).

The electrochemical behavior of (**1**) and (**2**) was studied in CH_2_Cl_2_ by cyclic voltammetry. This study is useful for determining the changes in the electronic density of ruthenium (II) and (III) upon coordination with HQ. The precursor complex (**P1**) exhibits an oxidation process corresponding to Ru^II^/Ru^III^, ranging from 300 to 400 mV [26,31]. After the coordination of HQ, the oxidation process Ru^II^/Ru^III^ increased considerably to 917.36 mV (Figure 4a). The higher oxidation potential observed for complex (**1**) compared with the precursor is due to the replacement of two chloride ligands, which are good donors of electronic density, by oxygen atoms, which are poor donors. This phenomenon is consistent with findings in the literature for other ruthenium complexes that exhibit the O-O chelating mode [31]. The results confirm that (**1**) presents a quasi-reversible process and stability after the coordination of the HQ ligand. For (**2**), an irreversible process (Ru^III^/Ru^II^) was observed at 40.36 mV, a higher value compared to (P2), which is −69.00 mV (Figure S7 of the Supplementary Materials). This indicates that the metallic center can be reduced more easily after the replacement of one Cl (σ/π donor ligand) and one H_2_O by HQ. The new ligand groups (carbonyl and phenolate) possibly have a lower electron density donor character due to the molecule’s highly conjugated system. This observation aligns with our previous work on Ru(III) complexes that present the O–O chelating mode [32].

**Figure 5:**
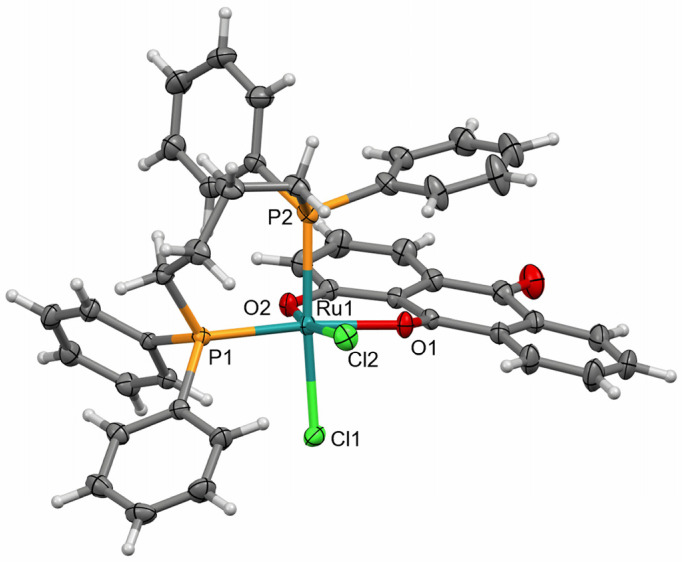
Ellipsoid representation (25% probability) of (**2**) showing its asymmetric unit with atom labeling scheme for the Ru coordination sphere. Hydrogen atoms are depicted as spheres of arbitrary radii.

Figure 5 shows the crystal structure ellipsoid representation of (**2**), which crystallizes in the monoclinic space group P2_1_/c. The crystal structure confirms an octahedral hexacoordinated complex with the cation bound to the two phosphorous atoms from the dppb ligand, two *cis*-positioned chloride anions, and the deprotonated HQ ligand, which is coordinated in a bidentate fashion. The chelation involves the carboxyl (O1) and hydroxyl (O2) oxygen atoms, *trans*-positioned to phosphorous (P1) and chloride (Cl2) atoms, respectively. 

**Figure 6:**
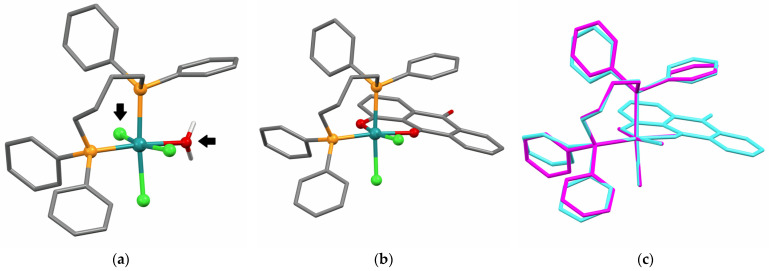
Capped sticks representations of (**a**) the precursor (**P2**) [27]; (**b**) the complex (**2**) structures highlighting the coordination replacement positions; and (**c**) the molecular superposition, matched by the homologous phosphorus and chloride atoms in the coordination sphere using the Mercury structure overlay calculation.

As expected, the HQ ligand replaced the water ligand and one of the chloride ligands *cis* to the dppb phosphorous atom in the precursor (**P2**) (Figure 6). The structural superposition of (**2**) and its precursor [27] highlights their intramolecular geometric similarities, considering the dppb and chlorine moieties (Figure 6). The stereochemistry and coordination sphere of (**2**) are very similar to the analogous structures containing Ru(III) complexes with hydroxybenzophenones with the general formula *cis*-[RuCl_2_(HB)(dppb)], obtained from the same precursor [32].

As expected, the Ru–P bond length (Ru-P2 = 2.372(1) Å) *trans* to chloride atom (higher σ-donor character) [40] is significantly longer than the Ru-P bond length (Ru-P1 = 2.296(1) Å) *trans* to oxygen atom due to the *trans* effects of the phosphorus atoms [32,41,42]. For the same reason, the Ru–Cl bond length (Ru-Cl1 = 2.378(1) Å) *trans* to phosphorus atom is slightly longer than the Ru–Cl bond length (Ru-Cl2 = 2.326(1) Å) *trans* to oxygen atom. The Ru–O1 bond length is slightly longer (2.101(2) Å) than the Ru–O2 bond (1.996(2) Å) due to their respective metal–carbonyl and metal–phenolate features [34,38]. Since the complex ligands do not possess classical hydrogen bond donors, the stabilization of the packing is governed by weak intermolecular interactions, such as non-classical hydrogen bonds and van der Waals forces.

Finally, the stability of the complexes in solution was evaluated in DMSO and in a mixture of DMSO-DMEM from 0 to 48 h using ^31^P{^1^H} NMR spectroscopy for (**1**) and UV–vis spectroscopy experiments for (**2**) due to its paramagnetic nature (Supplementary Materials: Figures S8–S11). The results showed that (**1**) formed byproducts after solubilization in DMSO; however, the major product remained the original complex after 48 h. Interestingly, when solubilizing (**1**) in the DMSO-DMEM mixture, smaller changes were observed. On the other hand, experiments with (**2**) showed that it was stable for 48 h in DMSO, but considerable changes were observed in the presence of the DMSO-DMEM mixture.

### 2.2. Electronic Properties, UV–Vis Absorption Spectra, and TD-DFT Calculations

**Figure 7:**
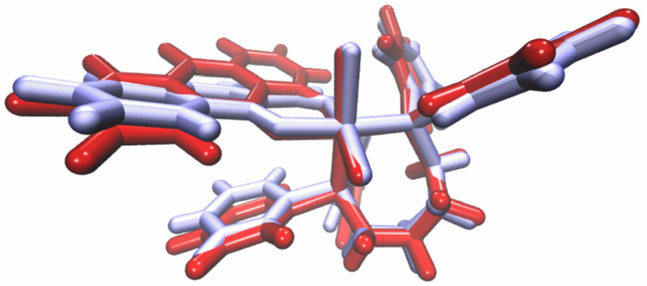
The superposition of structures obtained by X-ray diffraction (in blue) and calculated using the PBE0 functional (in red).

A comparative assessment of bond lengths and angles between PBE0 calculations and experimental data based on the geometry of (**2**) reveals low values for mean unsigned error (MUE) and mean absolute error (MAE), confirming the excellent agreement of the calculated geometry (Supplementary Materials: Table S1). This agreement can be visually confirmed through the superposition of both sets of geometries, as illustrated in Figure 7. Significant differences are observed in the HQ and phenyl dihedral angles, which is a common feature when comparing gas and condensed phases. Furthermore, other DFT methods were employed for comparative analysis, and the marginal variation among them indicates a limited sensitivity to the choice of DFT methodologies in calculating geometry. This trend aligns with observations in other metal complex systems [43,44,45,46].

**Figure 8:**
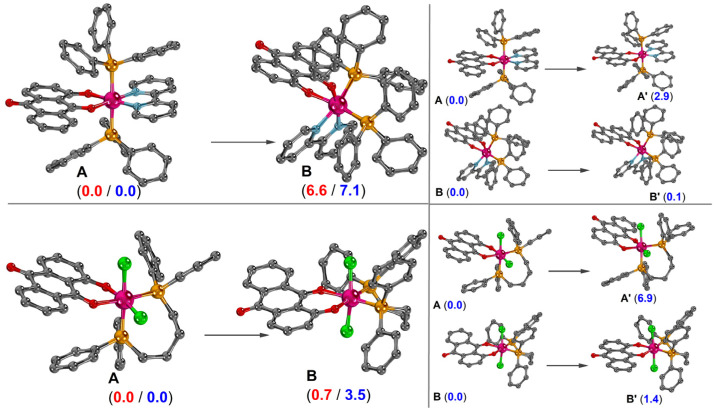
Comparison of relative Gibbs free energy (in kcal mol^−1^ at 298.15K and 1 atm; gas phase values in red and solvent values in blue) between the *trans-*(**A**) and *cis-*(**B**) arrangement of PPh_3_ ligands in (**1**) (**top right**) and the *cis-*(**A**) and *trans*-(**B**) configuration of chlorine atoms in (**2**) (**bottom right**). On the left side, the relative Gibbs free energy in solvent is shown for different HQ coordination modes (**A′** and **B′**) corresponding to each configuration of the ruthenium–HQ complexes. Hydrogen atoms in the structures were omitted for clarity.

Energetic analysis was conducted to shed light on the energy gap associated with configuration changes in both ruthenium complexes, with lyric **A** set for the conformation observed under experimental conditions (see Figure 8). Based on Gibbs free energy (at 298.15K and 1 atm), (**2**) exhibits a nearly 4 kcal mol^−1^ difference in the orientation of chlorine atoms, with solvent playing a significant role in the relative stability of the *cis-* against *trans-*arrangement. Conversely, for (**1**), the difference increases to 7 kcal mol^−1^ comparing *trans-* and *cis-*arrangements of PPh_3_ ligands. Remarkably, this variation is minimally affected by solvent. These conformational values involving chlorine and PPh_3_ ligands are in accordance with findings in other ruthenium compounds [32,47,48]. In addition, we explored a different coordination mode of HQ, assigning gaps ranging from 1.4 to 6.9 kcal mol^−1^ across the arrangements of both complexes, thus confirming the experimental results.

**Figure 9:**
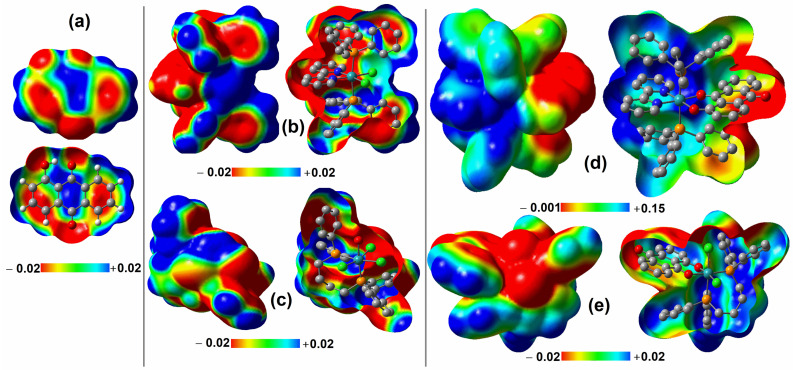
The molecular electrostatic potential (with an isovalue of 0.001 a.u.) of (**a**) HQ; (**b**) (**P1**); (**c**) (**P2**); (**d**) (**1**); and (**e**) (**2**). In these maps, regions in blue and red indicate positive and negative potentials, respectively. Each item includes a front view of the solid surface, and a bottom side view revealing the back of the surface and displaying the molecular disposition. Hydrogen atoms in the ruthenium structures were omitted for clarity.

Insights into the charge distribution were obtained through molecular electrostatic maps (see Figure 9). Within the HQ ligand, the negative areas in red are predominantly localized on oxygen atoms and lateral phenyl rings. When examining the precursor compounds, a distinct pattern was observed. In (**P2**), positive and negative areas are delocalized throughout the structure. In contrast, the negative regions in (**P1**) are centralized in aromatic structures, specifically in HQ and in the center of the phenyl rings of both PPh_3_ ligands.

The compound (**1**) displays a distinct separation between positive and negative regions oriented towards the metal-complex moieties. The negative regions are centrally clustered around the HQ ligand and the parallel phenyl rings. In contrast, (**2**) exhibits a different pattern, with negative regions concentrated within the coordination sphere and along the phenyl rings of the dppb ligand, as well as the aromatic structure of HQ. Additionally, when comparing (**2**) and its precursor using the GAPT method, there was only a negligible increase in ruthenium charge. This contrasts with (**1**), where a more pronounced increase in ruthenium charge was observed.

Turning attention to a combined analysis between TD-DFT calculations and UV–vis analysis, it can be observed that HQ exhibits two bands at 407 and 333 nm, mainly governed by π→π^*^ transitions and with minor contributions from oxygen p-orbitals (Supplementary Materials: Figure S12). One interesting aspect of these two bands is that the transition in 407 nm originates from the juglone moiety into the lowest unoccupied molecular orbital (LUMO), while the band at 333 nm arises from the benzene moiety (from the other side of the structure) into the LUMO. Although the band at 257 nm shares a similar nature with the other two bands, the intense band at 272 nm shows a different pattern, with transitions originating from carbon–carbon σ-orbitals and lone pair electrons of oxygen into the LUMO. On the other hand, in the ruthenium precursors, the UV–vis spectra show a predominant contribution from metal–ligand-to-metal–ligand charge transfer (MLMLCT) processes where chlorine atoms play a crucial role in the main transitions.

In (**1**), the band at 528 nm shows a dominant transition characterized by a mixed nature (Supplementary Materials: Table S2). This electronic transition shows metal–ligand charge transfer (MLCT) involving the charge transfer from the ruthenium d-orbital (Ru-d) into the HQ anti-ligand π-orbital (π*_HQ_) and a minor contribution from MLMLCT. The latter includes contributions from π_HQ_, bipy anti-ligand π-orbital (π*_bipy_), and both ruthenium ligand and anti-ligand d-orbitals. Moreover, similar MLCT and mixed MLCT/MLMLCT characters are observed for the experimental bands at 425 nm and 350 nm, respectively. In contrast, the intense band featuring peaks at 296 nm and 256 nm is marked by distinct contributions. In this region, the MLCT is based on Ru-d→π*_PPh3_ transitions accompanied by contributions from metal-to-metal–ligand charge transfer (MMLCT) and metal–ligand-to-ligand charge transfer (MLLCT).

In (**2**), it is crucial to note that chlorine atoms play a significant role in the majority of UV–vis transitions, displaying MLMLCT, MLLCT, ligand-to-metal–ligand charge transfer (LMLCT), and ligand-to-ligand charge transfer (LLCT) characteristics. The broad band with a peak at 668 nm demonstrates a mixed MLLCT/MLMLCT character, whereas the band at 341 nm is characterized by MLLCT. Conversely, the electronic transitions in the bands at 455 nm and 286 nm exhibit a more delocalized nature, contributing to a diverse range of MLLCT, MLMLCT, LLCT, and LMLCT contributions. It is important to note that both bands show contributions from the dppb ligand.

### 2.3. DNA-Binding: UV–Vis Spectrophotometric Titration

DNA is one of the main therapeutic targets studied against cancer because cell proliferation is controlled by this macromolecule [17,49]. Therefore, evaluating the interactions of complexes with DNA is a crucial criterion in the development of potential drugs [50]. In this context, the interactions between (**1**), (**2**), and DNA were evaluated by UV–visible absorption spectroscopy, and their binding constants (Kb) were determined.

**Figure 10:**
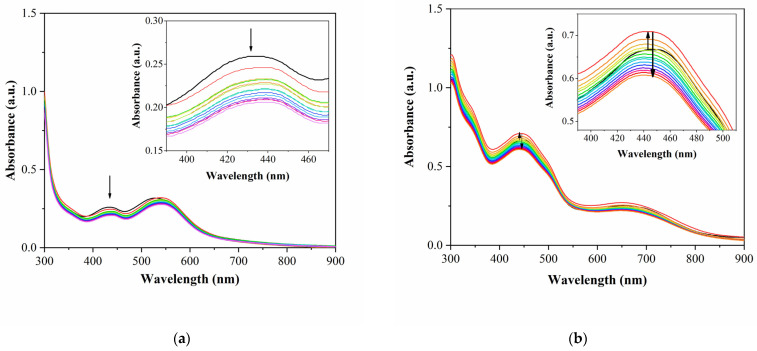
Changes in the UV–vis spectra of (**1**) and (**2**) with increasing concentrations of ct-DNA (0–8.20 × 10^−5^ mol L^−1^): (**a**) (**1**) [1] = 1.00 × 10^−3^ mol L^−1^ and (**b**) (**2**) [2] = 1.0 × 10^−3^ mol L^−1^ in Tris-HCl buffer (4.50 mM Tris HCl; 0.50 mM Tris base and 50.0 mM NaCl, pH 7.4) at 298 K. The insets show magnified views of the spectral ranges of the bands used to calculate the Kb values.

The UV–vis spectra obtained for (**1**) after increasing additions of ct-DNA (Figure 10a) demonstrated hypochromism and a shift of the bands to the red region of the spectrum (bathochromism). This effect is characteristic of the intercalative interaction mode of the complex with DNA [51,52,53,54,55], and similar behavior has been observed in other studies with complexes containing anthraquinone derivative ligands [56,57].

On the other hand, the initial additions of the ct-DNA solution to (**2**) (Figure 10b) showed an increase in absorbance (hyperchromism). As the ct-DNA concentration increased, a hypochromic effect was observed along with a very slight blue shift. The simultaneous effects of hyperchromism and hypochromism with DNA addition have been described for other hydroxyanthraquinones, such as quinizarin and danthron [58].

The Kb values were calculated from the absorption band at 430 nm for (**1**) and 445 nm for (**2**). The values obtained for the binding constant are summarized in Table 3, along with the percentage of hypochromism for each complex.

The compounds (**1**) and (**2**) exhibited Kb values on the order of 10^5^ M^−1^, demonstrating similar values to those of metal complexes that bind ct-DNA through non-covalent interactions (electrostatic or hydrogen bonding). Such interactions also occur with other Ru(II)/phosphinic/diimine complexes, as reported in the literature [41,59].

### 2.4. Cytotoxicity Analysis

**Figure 11:**
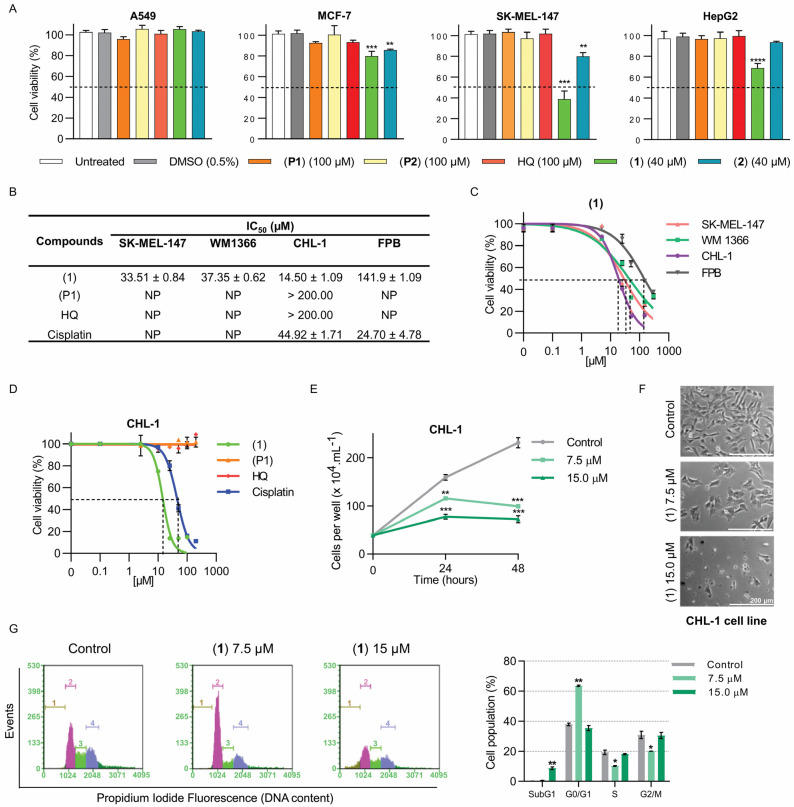
Compound (**1**) is cytotoxic to melanoma cells. (**A**) Screening data showing that melanoma cell SK-MEL-147 was more responsive to treatment with (**1**) than HepG2, A549, and MCF-7. The cells were treated with (**1**) at 40 µM for 48 h. (**B**) IC_50_ values (µM) were determined from dose–response curves obtained by MTS assay. Melanoma cells (SK-MEL-147, WM1366, and CHL-1), and FPB (dermal primary fibroblast) were treated with (**1**) or its precursors (**P1**) and HQ for 48 h. Cisplatin was used as a positive control. NP: not performed. (**C**,**D**) Dose–response curves determined by MTS. (**E**) Cell growth kinetics from 0 to 48 h were determined by counting viable cells in the presence of Trypan Blue. (**F**) Contrast microscopy images showing the morphological characteristics of CHL-1 cells treated with 7.5 and 15 µM of (**1**) for 24 h. The scale bar indicates 200 µm. (**G**) Representative histograms obtained from cell cycle analysis using flow cytometry. CHL-1 cells were treated for 24 h with (**1**) at 7.5 or 15 µM. Sub-G1 (brown, 1), G0/G1 (pink, 2), S (green, 3), G2/M (blue, 4). **** *p* < 0.0001, *** *p* < 0.001, ** *p* < 0.01, and * *p* < 0.05 compared to the control group, according to one-way analysis of variance (ANOVA) followed by Dunnett’s post-test.

The cytotoxic activity of (**1**) and (**2**) was screened on A549 (lung adenocarcinoma), MCF-7 (estrogen-positive breast cancer), SK-MEL-147 (melanoma), and HepG2 (hepatocellular carcinoma) cell lines using the MTS assay. Tumor cell cultures were treated with (**1**) and (**2**) at 40 µM, and (**P1**), (**P2**), and HQ at 100 µM for 48 h. Notably, (**1**) demonstrated significant cytotoxicity against the SK-MEL-147 cell line, reducing cell viability by approximately 60% (Figure 11A). Additionally, no significant cytotoxic effects were observed when the tumor cell lines were treated with (**P1**), (**P2**), or HQ (Figure 11A). These data demonstrate that the chemical coordination involved in the formation of the complexes was essential for their cytotoxic activity.

Given the significant cytotoxic effects of (**1**) on SK-MEL-147 melanoma cells, we included other melanoma-derived cell lines (CHL-1 and WM1366), considering that malignant melanomas are highly heterogeneous depending on their genetic background [60,61]. Cell lines with different mutation profiles are useful as study models and help us understand the possible mechanism of action involved in the antitumor potential of the studied prototype [62]. While (**1**) was active against all three cell lines, its potency was higher on CHL-1 (IC_50_ of 14.50 ± 1.09 µM) compared to SK-MEL-147 (IC_50_ = 33.51 ± 0.84 µM) and WM1366 (IC_50_ = 37.35 ± 0.62 µM) (Figure 11B). Interestingly, dermal primary fibroblasts (FPB) were less affected by treatment with (**1**) (IC_50_ of 141.9 ± 1.09 µM). These findings align with other studies showing the high selectivity of ruthenium complexes toward tumor cells [59,63,64]. Importantly, the cytotoxicity of (**1**) was much more pronounced (around three times higher) on CHL-1 compared to cisplatin, and its selectivity was approximately five times higher than cisplatin (Figure 11C,D). Our data are corroborated by other studies that showed superior antitumor potential of the ruthenium complexes compared to cisplatin [65,66].

We also observed changes in the proliferative behavior of CHL-1 cells treated with (**1**), suggesting the antiproliferative activity of this compound on melanoma cells. Counting the viable cells at 24 and 48 h revealed that (**1**) inhibited the growth of CHL-1 cells (Figure 11C). The frequency of viable cells was reduced to 27.22% and 71.29% after 24 and 48 h of treatment, respectively, when (**1**) was used at 7.5 μM (Figure 11E). In cultures treated with (**1**) at 15 μM, the frequencies of viable cells were 48.73% (24 h) and 20.80% (48 h) (Figure 11E). Morphological changes in CHL-1 cells were observed after 24 h of treatment. Treated CHL-1 cells exhibited irregular shapes as well as the presence of cytoplasmic vacuoles (Figure 11F).

We also performed cell cycle analysis using flow cytometry to determine whether the alterations in cell growth kinetics promoted by (**1**) are related to its ability to inhibit cell cycle progression. Interestingly, the analyses revealed that (**1**) induced cell cycle arrest at the G1/S transition. Samples treated with (**1**) at 7.5 µM for 24 h showed a substantial increase in the G0/G1 cell population, accompanied by a decrease in the frequency of cells in the S-phase and G2/M-phases (Figure 11G), thereby effectively inhibiting cell progression.

The coordinated regulation of the cell cycle is essential for maintaining cellular homeostasis [67]. G0/G1 arrest is a crucial response to DNA damage caused by various factors, including oxidative stress. Thus, cell cycle arrest acts as a protective mechanism to give the cells time to repair DNA lesions. However, when DNA damage is too extensive and cell cycle arrest persists for a prolonged period, the cells may undergo apoptosis [68,69]. In our previous study, the treatment with (**1**) resulted in a notable G0/G1 arrest, which can be attributed to increased expression levels of cyclin-dependent kinase inhibitors (CDKIs), such as p21 or p27. These inhibitors can block the activity of cyclin–CDK complexes, preventing the G1/S or G2/M transitions [70,71,72,73].

Furthermore, a recent study demonstrated that a ruthenium(II) complex coordinated with cinnamic acid also induced G1/S transition arrest, associated with an increase in CDKN1A (p21) expression and alterations in CCND1 (cyclin D) and CCNE2 (cyclin E) [74]. These findings align with our results, as the antiproliferative effects of (**1**) on CHL-1 were accompanied by cell cycle arrest at the G1/S transition. Further studies will be performed to investigate the influence of (**1**) on the regulation of cyclins and their inhibitors.

**Figure 12:**
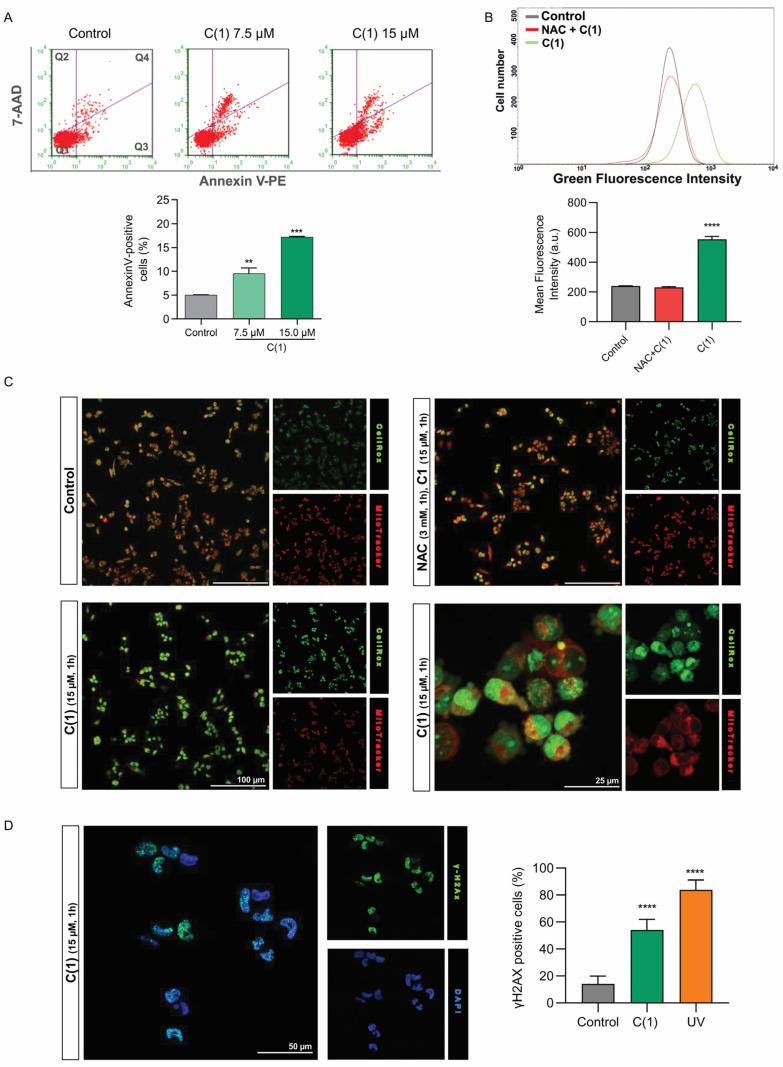
Compound (**1**) induces apoptosis via ROS generation in the CHL-1 cell line. (**A**) Representative dot plot graphs obtained from the Annexin V/7AAD analysis using flow cytometry. Q1, viable cells; Q2 necrotic cells; Q3, early apoptosis; Q4, late apoptosis. (**B**,**C**) Measurement of reactive oxygen species (ROS) levels by CellRox Green reagent using flow cytometry and confocal microscopy, respectively. CHL-1 cell was treated with (**1**) for 1 h. Representative histogram shows the control group (DMSO, black line), cells pre-treated with NAC (1 h) and exposed to (**1**) at 15 µM (red line), and cells treated with (**1**) at 15 µM (green line). (**D**) Illustrative fluorescence images obtained by confocal microscopy and phospho-H2AX (γ-H2AX) quantification, a DNA damage marker. The CHL-1 cell line was treated with (**1**) at 15 μM for 1h. Nuclei were stained with DAPI. Cells irradiated with ultraviolet C light for 20 min were used as positive controls. **** *p* < 0.0001, *** *p* < 0.001, and ** *p* < 0.01 vs. control group, according to ANOVA followed by Dunnett’s post-test.

The accumulation of cells in suspension and cellular debris in samples treated with (**1**), particularly at 15 µM (Figure 11F), along with the increased sub-G1 population found in cell cycle analysis (Figure 11G), indicates that (**1**) treatment may lead to cell death. Thus, the pro-apoptotic effect of (**1**) was investigated through the annexin V/7AAD assay using flow cytometry. (**1**) significantly increased the frequency of annexin V-positive cells in both 7.5 (~2-fold) and 15 µM (~3.5-fold) compared to control groups (Figure 12A).

Ruthenium-based complexes have been shown to promote cell cycle arrest and apoptosis in several cancer cell lines, highlighting their potential as therapeutic agents. For instance, Bomfim et al. [75] demonstrated that ruthenium(II) complexes with 6-methyl-2-thiouracil effectively inhibited cell proliferation and induced apoptosis in HL-60 cells (human acute promyelocytic leukemia). This process involved the activation of caspases, mitochondrial dysfunction, DNA damage, and the activation of the JNK/p38 pathways, contributing to the apoptotic response. Similarly, Costa et al. [76] showed that the ruthenium(II) complex 3-hydroxy-4-methoxybenzoate exhibits cytotoxic effects against A549 adenocarcinoma lung cancer cells. The processes involved the generation of reactive oxygen species (ROS) that culminated in several effects, including reduced cellular proliferation, cell morphology changes with actin cytoskeleton reorganization, cell cycle arrest at G2/M, apoptosis, changes in mitochondrial membrane potential, and DNA damage.

The mechanism often involved in the pro-apoptotic activity of ruthenium complexes is associated with increased production of reactive oxygen species (ROS) and oxidative stress [77]. Elevated ROS levels are associated with genomic instability and can trigger cell cycle checkpoints and apoptotic pathways [78,79,80]. To explore the connection between oxidative stress and the pro-apoptotic and antiproliferative activity of (**1**), we measured ROS intracellular levels using the CellRox^®^ Green oxidative stress reagent. CHL-1 cultures were treated with (**1**) at 15 µM for 1 h and analyzed by flow cytometry and confocal microscopy (Figure 12B,C). The results revealed higher ROS levels in cells treated with (**1**) compared to control cells. Importantly, when we pre-treated the cultures with the antioxidant N-acetyl-L-cysteine (NAC) before exposing them to (**1**) treatment for 1 h, the release of ROS was reversed. These findings indicate that the cytotoxic activity of (**1**) on CHL-1 is possibly due to its pro-oxidant effect.

It is well-documented that oxidative stress may promote DNA damage [81,82,83,84], and to evaluate whether (**1**) was able to induce DNA damage in CHL-1 cells, we assessed the frequency of cells positive for phospho-H2AX (γ-H2A.X), a well-established marker of DNA damage. The results showed an increased frequency of γ-H2A.X-positive cells in samples treated with (**1**) at 15 μM for 1 h (Figure 12D). Thus, our findings suggest that the enhanced cytotoxicity of (**1**) on melanoma cells is likely due to its ability to generate ROS, induce DNA damage, cause cell cycle arrest, and ultimately promote apoptosis. Our findings support further investigation to explore the molecular targets involved in the antitumor potential of (**1**) and its long-term effects.

## 3. Materials and Methods

### 3.1. Chemicals

Solvents were purified by standard methods, and all manipulations involving solutions were performed under an argon atmosphere using a Schlenk line. All chemicals used were of reagent grade or comparable purity. RuCl_3_∙xH_2_O and ligands 1-hydroxy-9,10- anthraquinone, 2,2′-bipyridine, triphenylphospine, and 1,4-bis(diphenylphosphino)butane were used as received from Sigma-Aldrich™. The precursors (**P1**) and (**P2**) were synthesized following the procedures outlined in the literature references [26,27], respectively.

### 3.2. Experimental

Elemental analyses were performed using a Fisons EA 1108 model (Thermo Scientific^TM^, Waltham, MA, USA). Conductivity measurements were obtained at 298 K in CH_2_Cl_2_ containing 1 × 10^−3^ mol L^−1^ of the complexes using an mCA-100 model conductivity meter (MS Tecnopon^TM^, Piracicaba, SP, Brazil). FTIR spectra of the complexes were recorded using KBr pellets with a Shimadzu-IR-Prestige-21 spectrophotometer in the range of 400–4000 cm^−1^, with a resolution of 4 cm^−1^ and 32 scans. UV–vis spectra were recorded in CH_2_Cl_2_ solution, scanning from 190 at 850 nm, using an Evolution 60S model spectrophotometer (Thermo Scientific^TM^) and a quartz cuvette with a 1 cm path length. Cyclic voltammetry (CV) experiments of the complexes were conducted using an Autolab^TM^ PGSTAT 128N (Eco Chemie^TM^, Utrecht, The Netherlands) potentiostat-galvanostat under an argon atmosphere. These experiments were carried out at room temperature in CH_2_Cl_2_ containing 0.10 mol L^−1^ Bu_4_N^+^ClO_4_^-^ (Sigma-Aldrich^TM^) as the supporting electrolyte, using a one-compartment cell with both working and auxiliary electrodes as stationary Pt foils, and an Ag/AgCl reference electrode. The NMR experiments of (**1**) were conducted on a BRUKER^TM^ 300 MHz (Berlin, Germany) instrument using a BBO 5 mm probe at 298 K, with TMS as an internal reference. For ^1^H, ^13^C{^1^H}, and ^31^P{^1^H} NMR spectra, CDCl_3_ was used as the solvent. The splitting of proton, carbon, and phosphorus resonances was reported as s = singlet, d = doublet, t = triplet, and m = multiplet.

### 3.3. Synthesis and Characterization

#### 3.3.1. Synthesis of (**1**)

In a Schlenk flask containing 15 mL of degassed ethanol, 13.0 mg (0.0586 mmol) of HQ was dissolved, and the ligand was deprotonated with 50 µL of triethylamine. Then, 50.0 mg (0.0586 mmol) of (**P1**) was dissolved in 15 mL of dichloromethane and added to the reaction mixture. Subsequently, 19.0 mg (0.1172 mmol) of NH_4_PF_6_ was added to induce precipitation. The reaction mixture was refluxed and stirred under an argon atmosphere until the formation of a dark purple precipitate. The solid was filtered off, washed with water and hexane, and dried under *vacuum*.

#### 3.3.2. Data for (**1**)

Yield: 46.70 mg (69%). Anal. Calc. for C_60_H_45_F_6_N_2_O_3_P_3_Ru: exp. (calc.) C, 62.86 (62.66); H, 3.96 (3.94); N, 2.32 (2.44). ^31^P{^1^H} NMR (130 MHz, CDCl_3_, 298 K): δ(ppm) 25.39 (s). ^1^H NMR (300 MHz, CDCl_3_, 298 K): δ (ppm) 9.35–9.27 (dd, 2 H aromatic hydrogen for bipy); 8.22–8.19 (d, 1 H), 8.09-8.02 (m, H5, H8 of HQ); 7.77–6.95 overlapped signals, 40 H aromatic hydrogen for PPh_3_, bipy and HQ. ^13^C NMR (75 MHz, CDCl_3_, 298 K): δ(ppm) 170.56 (C1-O of HQ), 182.92 (C9=O of HQ), 178.18 (C10=O of HQ). UV–vis (CH_2_Cl_2_, 1.27 × 10^−5^ mol L^−1^): λ/nm (Ɛ/M^−1^ L cm^−1^) 252 (81.889), 296 (44.204), 350 (shoulder), 425 (shoulder), and 528 (18.535).

#### 3.3.3. Synthesis of (**2**)

In a Schlenk flask containing 10 mL of previously degassed methanol, 17.0 mg (0.0767 mmol) of HQ was dissolved, and the ligand was deprotonated with an equimolar amount of NaOH (0.1 mol L^−1^ water solution). Then, 50.0 mg (0.0767 mmol) of the precursor (**P2**) was dissolved in 10 mL of dichloromethane and added to the reaction mixture. The reaction was kept under an inert atmosphere and stirred for 24 h at room temperature. The solution was reduced under *vacuum* until all dichloromethane was removed, forming a green precipitate in methanol, which was filtered off, washed with water, and dried under *vacuum.*

#### 3.3.4. Data for (**2**)

Yield: 49.20 mg (78%). Anal. Calc. for C_42_H_35_Cl_2_O_3_P_2_Ru: exp. (calc.) C, 61.68 (61.39); H, 4.27 (4.29). UV–vis (CH_2_Cl_2_, 1.63 × 10^−5^ mol L^−1^): λ/nm (Ɛ/M^−1^ L cm^−1^) 248 (71.552), 286 (shoulder), 341 (shoulder), 455 (10.847), and 668 (5.859).

### 3.4. X-Ray Crystallography

Single crystals of (**2**) were obtained from the mother solution using the slow evaporation solution growth technique. Well-shaped single crystals were chosen for the X-ray diffraction experiment, which was performed with a Rigaku^TM^ (Tokio, Japan) XtaLAB mini II diffractometer (graphite monochromator) using Mo-Kα radiation (λ = 0.71073 Å). The programs SAINT and SADABS [85] and CrysalisPro [86] were used for data collection, cell refinement, data reduction, and multi-scan method absorption correction. The structure was solved and refined using the software SHELX-2018/3 [87]. Structure analysis and artwork preparation were performed with Mercury software [88]. All atoms, except hydrogen, were clearly identified and refined by least squares full matrix F^2^ with anisotropic thermal parameters. The main crystal data collection and structure refinement parameters for (**2**) are summarized in Table 4.

### 3.5. Computational Methodology

All the calculations were performed using the Gaussian09 package [89]. We employed the Perdew–Burke–Ernzerhof–Adamo functional (PBE0, also known as PBE1PBE) [90,91] for both optimization and the characterization of electronic properties, including TD-DFT calculations [92,93,94]. Closed-shell systems, namely HQ, (**P1**), and (**1**), were investigated using the restricted PBE0 functional. In contrast, open-shell systems, including (**P2**) and (**2**), were analyzed with the unrestricted PBE0 functional. Furthermore, single-point restricted and unrestricted TD-PBE0 calculations were performed on closed- and open-shell systems, respectively. It is important to note that PBE0 is a well-established functional known for its accuracy in describing metal complexes [95,96]. The def2-TZVP basis set was applied to the ruthenium atom, while def2-SVP was used for other chemical elements [97]. The integral equation formalism version of the Polarizable Continuum Model (IEF-PCM) [98] was used to account for the CH_2_Cl_2_ solvation effect, and the generalized atomic polar tensor (GAPT) [99] method was used to evaluate atomic charge distribution. Additional software tools were also employed, such as GaussView5.0.8 [100] for plotting the molecular electrostatic potential (MEP), Iboview [101] for illustrating isomers, and VMD [102] for the analysis of structural superpositions. Moreover, it is noteworthy that additional DFT methods were applied for comparative purposes, including B3LYP [103,104], B97D [105], M06 [106], and M06L [107]. 

### 3.6. DNA Binding Studies

UV–vis studies were performed using a Shimadzu^TM^ UV-1800 (Kyoto, Japan) spectrophotometer at room temperature to investigate the interaction of the complexes with ct-DNA (calf thymus DNA). A standard solution of ct-DNA from Sigma-Aldrich^TM^ was prepared by diluting about 20 mg of ct-DNA in 10 mL of Tris-HCl buffer (4.50 mM Tris HCl; 0.50 mM Tris base and 50.0 mM NaCl, pH 7.4). Subsequently, 2000.0 μL of buffer and 80.0 μL of ct-DNA solution were added to a quartz cuvette, and its concentration was determined by UV–vis spectroscopy. The exact concentration of ct-DNA was determined from its UV absorbance at 260 nm (ɛ = 6600 M^−1^ cm^−1^) [108]. The experiments were conducted by keeping the concentration of the complexes constant and titrating 20 µL of ct-DNA solution into the sample. The absorption wavelengths were recorded in the range of 190–900 nm after each titration. The intrinsic equilibrium binding constant (Kb) of the complexes to ct-DNA was obtained by monitoring the changes in the absorption intensity with increasing concentration of ct-DNA and analyzed by regression analysis using the neighbor exclusion equation.

### 3.7. Biological Assays

#### 3.7.1. Cell Lines and Culture Conditions

The A549, MCF-7, HepG2, and melanoma cells (SK-MEL-147, CHL-1, and WM1366) were used in this study. The melanoma cells were kindly provided by Dr. Roger Chammas from Instituto do Cancer do Estado de São Paulo (ICESP), while the other cell lines were acquired from the Rio de Janeiro Cell Bank (RJCB). The cell lines were authenticated through RJCB’s service. Cells were cultured in Dulbecco’s modified Eagle’s medium (DMEM)/F12, (catalog no. D8900, Sigma-Aldrich, Saint Louis, MO, USA) supplemented with 10% fetal bovine serum (FBS, Cultilab, SP, Brazil). The cultures were maintained in an incubator at 37 °C with a controlled atmosphere (95% air and 5% CO_2_), and subculturing was performed regularly every 2 or 3 days.

Primary fibroblasts were established from 1.5 × 1.5 mm fragments of skin (FPB) using DMEM/F12 culture medium supplemented with 10% FBS, penicillin/streptomycin (50 units/50 μg/mL, Thermo Scientific, Wilmington, DE, USA) and amphotericin B (2.5 μg/mL, Thermo Scientific). The biological material was collected following the rules established by the Human Research Ethics Committee (#69453817.9.0000.5142) by Dr. Angel Mauricio Castro Gamero from UNIFAL-MG.

#### 3.7.2. Cell Viability Evaluation

##### Colorimetric Assay Based on the Conversion of Tetrazolium Salt to Formazan (MTS)

Cells were seeded in 96-well plates at a density of 1 × 10⁴ cells per well. Firstly, (**1**), (**2**), (**P1**), (**P2**), and HQ were screened at concentrations of 40 and 100 µM for 48 h. Four cancer cell lines (A549, HepG2, MCF-7, and SL-MEL-147) were tested to identify the most active complex. In subsequent assays, these compounds were tested at different concentrations (0.1, 1, 10, 25, 50, and 100 µM) for 48 h using melanoma cell lines and primary fibroblasts. Cell viability was assessed using CellTiter 96^®^ AQueous One Solution Cell Proliferation Assay (catalog no. #G3580, Promega, Madison, WI, USA) according to the manufacturer’s instructions. Following 4 h of incubation at 37 °C, the samples were analyzed using a spectrophotometer at 490 nm. IC_50_ values, representing the concentration required to inhibit 50% of cell growth, were calculated using Calcusyn software (Biosoft, Ferguson, MO, USA).

##### Trypan Blue Exclusion Test

Cells were seeded on 35 mm plates at a density of 2 × 10^5^ cells/plate. After 24 h, the cells were treated with (**1**) at concentrations of 7.5 and 15 µM for 24 or 48 h. Viable and non-viable cells were then scored using a hemocytometer in the presence of 0.4% Trypan Blue dye, observed under an inverted phase contrast microscopy (Zeiss, Oberkochen, Germany). 

#### 3.7.3. Cell Cycle Analysis

Cells were seeded in 35 mm plates at a density of 2 × 10^5^ cells/well. After adhesion, the cells were treated with (**1**) at concentrations of 7.5 and 15 µM for 24 h. Following treatment, the cells were harvested using a trypsin-EDTA solution (Sigma-Aldrich). The samples were centrifuged (5 min at 300× *g*) and cells were fixed with ethanol (75% in PBSA) at 4 °C for 24 h. After another centrifugation cycle, the cells were stained for 1 h (PBSA, RNase 1.5 mg/mL, and propidium iodide 90 µg/mL. The analysis was performed using a flow cytometer (Guava easyCyte 8HT, Hayward, CA, USA) with GuavaSoft 2.7 software. The DNA content of 10,000 cells was scored per sample, and the amount of DNA per cell was directly proportional to the fluorescence emitted by propidium iodide. Thus, the cell populations distributed in different phases of the cell cycle were determined.

#### 3.7.4. Annexin V/7-AAD Assay

Apoptosis was assessed using the Guava Nexin Reagent Kit (catalog no.4500-0450, Merk Millipore, Burlington, MA, USA) following the manufacturer’s instructions. Briefly, cells were seeded in 35 mm plates at a density of 2 × 10^5^ cells per well. After adhesion, the cells were treated with (**1**) at 7.5 and 15 µM for 24 h. Then, the samples were collected (trypsin-EDTA, Sigma-Aldrich), centrifuged at 300× *g* for 5 min, washed in PBSA, and homogenized in a solution containing Annexin V conjugated to PE and 7-AAD. After 20 min, the samples were analyzed using a flow cytometer (Guava easyCyte 8HT) with GuavaSoft 2.7 software (5000 events were acquired).

#### 3.7.5. Measurement of Reactive Oxygen Species (ROS) Levels

Intracellular ROS levels were detected using the CellRox Green^®^ reagent according to the manufacturer’s instructions. Cells were seeded in 35 mm plates at a density of 2 × 10^5^ cells per plate. After adhesion, the subconfluent cultures were treated with (**1**) at 15 µM for 1 h. Cells were then harvested and incubated in complete medium containing CellRox (catalog no. C10444, Thermo Fisher Scientific, Waltham, MA, EUA, 2.5 µM) at 37 °C for 30 min. The analysis was performed by flow cytometry (Guava easyCyte 8HT) using GuavaSoft 2.7 software (10,000 events were acquired). For fluorescence microscopy, cells were seeded in 35 mm plates on coverslips at a density of 2 × 10^5^ cells per plate. After allowing for adhesion, the cultures were treated with (**1**) at 15 µM for 1 h or pre-treated with N-acetyl-L-cysteine (NAC) (catalog no. A9165, Sigma, 3 mM) for 1 h before (**1**) treatment (15 µM, 1 h). Subsequently, the cells were incubated for 30 min in complete medium containing MitoTracker Red^®^ (catalog no. M22425, Thermo Fisher Scientific, Waltham, MA, USA, 500 nM), a cell-permeant fluorescent dye that stains active mitochondria in live cells, and CellRox Green Oxidative Stress (1 μM), a cell-permeant dye that exhibits bright green photostable fluorescence upon oxidation by reactive oxygen species (ROS), followed by two washes with PBS. The fluorescence images were immediately captured by a laser scanning confocal microscope (Nikon, Melville, NY, USA).

#### 3.7.6. DNA Damage Evaluation Through γ-H2A.X Immunodetection

The immunocytochemistry for phospho-H2A.X (γ-H2A.X) was performed following the protocol proposed by Noubissi et al. [109]. Cells were seeded in 35 mm plates on coverslips at a density of 1 × 10^5^ cells. After adhesion, the cells were maintained in a serum-free medium for 18 h and treated with (**1**) (15 μM) for 1 h. For the positive control, CHL-1 cells were irradiated with ultraviolet C light (5W, 20 min). The samples were then fixed with 3.7% formaldehyde for 15 min and treated with Triton X-100 (0.25%) for 10 min. After blocking with 3% albumin for 2 h, the cells were incubated with anti-phospho H2A.X (serine 139) (catalog no. 9718, Cell Signaling, Danvers, MA, 1:500) at 4 °C for 2 h. Subsequently, anti-rabbit secondary antibody–Alexa Fluor 488 conjugate (catalog no. ab150073, Abcam, 1:200) was added for 1 h at room temperature. Finally, the slides were mounted with Fluoroshield (catalog no. F6057, Sigma) and analyzed under a laser scanning confocal microscope (Nikon, Melville, NY, USA). A total of 500 cells were scored per slide.

#### 3.7.7. Statistical Analysis

The data are presented as mean ± standard deviation (SD) or mean ± standard error of the mean (SEM) from at least three independent experiments conducted in triplicate unless stated otherwise. The results were subjected to a one-way analysis of variance (ANOVA) followed by Dunnett’s post-test using GraphPad Prism^®^ software 8.0 (GraphPad Software, Inc., San Diego, CA, USA).

## 4. Conclusions

In summary, new ruthenium complexes with HQ were synthesized and characterized using various techniques. The interaction studies with ct-DNA presented binding constants of the order of 10^5^ M^−1^, comparable to values obtained with other ruthenium complexes. These results and the spectral changes observed for (**1**) and (**2**) suggest interaction with DNA through intercalation, electrostatic attraction, or hydrogen bonding. The cytotoxicity of both complexes was evaluated in tumor cell lines from human melanoma cancer (SK-MEL-147) and (**1**) was the most responsive. Thus, (**1**) was further evaluated in two other melanoma-derived cell lines (CHL-1 and WM1366) yielding very promising results in the CHL-1 cell line (CI_50_ = 14.50 μM ± 1.09) compared to cisplatin (reference drug) (CI_50_ = 44.92 ± 1.71). Furthermore, (**1**) was more cytotoxic than the free HQ and precursor (**P1**). Compound (**1**) also inhibited the proliferation of CHL-1 cells and induced apoptosis, with these events being associated, at least in part, with the inhibition of cell cycle progression at the G1/S transition due to the pro-oxidant effect of the complex. Therefore, this study reports a complex with promising antitumor and antimetastatic properties that may encourage future studies in the treatment of skin cancer.

## Figures and Tables

**Table 1:** Infrared frequencies (cm^−1^) of the HQ, (**1**), and (**2**).

	ν(C=O)	ν(C-O)	ν(P-F)	δ(P–F)	ν(Ru–P)	ν(Ru–O)	ν(Ru–N)
HQ	1672; 1636	1290					
(**1**)	1649; 1585	1306	839	557	518; 513	492	461
(**2**)	1662; 1611	1298			511; 496	476	

**Table 2:** ^13^C {^1^H} chemical shifts (ppm) for HQ and (**1**).

	C1	C2	C3	C4	C5	C6	C7	C8	C9	C10	C11	C12	C13	C14
HQ	162.90	124.69	137.08	119.88	127.24	134.51	135.00	127.75	188.99	182.72	133.53	133.95	116.48	133.79
(**1**)	170.56	124.77	135.93	119.26	126.12	134.51	134.88	126.69	182.92	178.18	133.49	133.62	118.42	133.56
Δδ	−7.66	−0.08	+1.15	+0.62	+1.12	0	+0.12	+1.06	+6.07	+4.54	+0.04	+0.33	−1.94	+0.23

**Table 3:** Binding constants (Kb) and hypochromism percentage (%H) of (**1**) and (**2**).

Complexes	Kb (10^5^ L mol^−1^)	%H
(**1**)	6.93	14.84
(**2**)	1.60	13.70

**Table 4:** Crystal data and structure refinement for (**2**).

	(2)
CCDC deposit number	2403244
Empirical formula	C_42_H_35_Cl_2_O_3_P_2_Ru
Formula weight	821.61
Temperature (K)	293(2)
Wavelength (Å)	0.71073
Crystal system	Monoclinic
Space group	P2_1_/c
Unit cell dimensions (Å, °)	a = 12.7580(6), α = 90 b = 10.1642(4), β = 94.237(4) c = 27.9431(10), γ = 90
Volume (Å^3^)	3613.6(3)
Z	4
Calc. density (g cm^−3^)	1.510
Absorption coef. (mm^−1^)	0.710
F(000)	1676.0
θ range (°)	2.565 to 27.103
Index ranges	−16 ≤ h ≤ 14 −13 ≤ k ≤ 13 −35 ≤ l ≤ 35
Reflections collected	39,931
Independent reflections	7,953 [R_int_ = 0.0361]
Completeness to θ_max._ (%)	
Data/restraints/parameters	7,953/0/451
Goodness-of-fit on F^2^	1.147
Final R indices [I > 2σ(I)]	R_1_ = 0.0343, wR_2_ = 0.0704
R indices (all data)	R_1_ = 0.0528, wR_2_ = 0.0809
Largest diff. peak and hole (e Å^−3^)	0.60 and −0.33

## Data Availability

All additional data analyzed during this study can be found in the “Supplementary Materials” section of this article.

## Supplementary Materials

The following supporting information can be downloaded at https://www.mdpi.com/article/10.3390/ph18010063/s1, Figure S1: Infrared spectrum for HQ, in KBr pellet; Figure S2: Infrared spectrum for (1), in KBr pellet; Figure S3: Infrared spectrum for (2), in KBr pellet; Figure S4: 1H NMR spectrum for HQ, in CDCl3; Figure S5: 13C{1H} NMR spectrum for HQ, in CDCl3; Figure S6: 13C{1H} NMR spectrum for (1), in CDCl3; Figure S7: Cyclic voltammogram of (P2) (TBAP 0.1 M; CH2Cl2; Ag/AgCl; work electrode Pt; 100 mV s−1); Figure S8: 31P{1H} NMR spectroscopy of (1): 0 min., 24 h and 48 h, in DMSO (D2O capillary); Figure S9: 31P{1H} NMR spectroscopy of (1): 0 min., 24 h and 48 h, in DMSO-DMEM mixture (D2O capillary); Figure S10: UV-vis spectra of (2): 0 min., 24 h and 48 h, in DMSO; Figure S11: UV-vis spectra of (2): 0 min., 24 h and 48 h, in DMSO-DMEM mixture; Figure S12: Experimental transitions (λexp, in nm) to each band detected in the HQ UV-Vis spectrum, along with computed PBE0 excitation energies (λ_calc_, in nm) and associated oscillator strength (*F*, in a.u.), are provided. Additionally, the contribution from the charge transfer process (in %) to the calculated electronic transition, involving occupied and unoccupied orbitals, is included. An isovalue of 0.03 a.u. was applied to the electronic density of the orbitals; Table S1: The mean absolute error (MAE) and the mean unsigned error (MUE) between the experimental and calculated values, involving bond lengths (in Å), angles (in degrees), and dihedral angles (in degrees), were assessed using different DFT methods employing the same basis sets; Table S2: Experimental electronic transition (λ_exp_, in nm) and computed PBE0 excitation energies (λ_calc_, in nm) with associated oscillator strength (*F*, in a.u.) of (**1**).
