# Supplementary material for: Ruthenium(II) Complex with 1-Hydroxy-9,10-Anthraquinone Inhibits Cell Cycle Progression at G0/G1 and Induces Apoptosis in Melanoma Cells
Source: Pharmaceuticals (Basel). 2025 Jan 8;18(1):63. doi: 10.3390/ph18010063 (PMC11768811; doi:10.3390/ph18010063)
Supplement: Supplementary file 1 [file pharmaceuticals-18-00063-s001.zip › pharmaceuticals-3372171-supplementary.pdf]

Ruthenium(II) Complex with 1-Hydroxy-9,10-Anthraquinone Inhibits Cell Cycle Progression at G0/G1 and Induces Apoptosis in Melanoma Cells

Júlia S. M. Dias [1], Guilherme A. Ferreira-Silva [2], Rommel B. Viana [3], João H. de Araujo Neto [4], Javier Ellena [5], Rodrigo S. Corrêa [6], Marília I. F. Barbosa [1], Marisa Ionta [2,*] and Antônio C. Doriguetto [1,*]

[1] Instituto de Química, Universidade Federal de Alfenas (UNIFAL-MG), Alfenas 37130-000, MG, Brazil; scaffjulia@gmail.com (J.S.M.D.); mariliaifrazaob@gmail.com (M.I.F.B.)

[2] Departamento de Ciências Biomédicas, Universidade Federal de Alfenas (UNIFAL-MG), Alfenas 37130-000, MG, Brazil; alfer.guilherme@gmail.com (G.A.F.S.)

[3] Departamento de Química, Universidade Estadual do Ceará (UECE), Limoeiro do Norte 62930-000, CE, Brazil; rommel.viana@uece.br

[4] Instituto de Química, Universidade de São Paulo (USP), São Paulo 05508-000, SP, Brazil; joaohonorato@iq.usp.br

[5] Instituto de Física de São Carlos, Universidade de São Paulo (USP), São Carlos 13566-590, SP, Brazil; javiere@ifsc.usp.br

[6] Departamento de Química, Universidade Federal de Ouro Preto (UFOP), Ouro Preto 35400-000, MG, Brazil; rodrigocorrea@ufop.edu.br

* Correspondence: marisa.ionta@unifal-mg.edu.br (M.I.); doriguetto@unifal-mg.edu.br (A.C.D.)

Supporting Information

Figure S1. Infrared spectrum for HQ, in KBr pellet.

Figure S2. Infrared spectrum for (**1**), in KBr pellet.

Figure S3. Infrared spectrum for (**2**), in KBr pellet.

Figure S4. ^{1}H NMR spectrum for HQ, in $CDCl_3$.

Figure S5. ^{13}C˚^{1}H} NMR spectrum for HQ, in $CDCl_3$.

Figure S6. ^{13}C˚^{1}H} NMR spectrum for (**1**), in $CDCl_3$.

Figure S7. Cyclic voltammogram of (**P2**) (TBAP 0.1 M; CH_2Cl_2; Ag/AgCl; work electrode Pt; 100 mV s^{-1}).

Figure S8. ^{31}P˚^{1}H} NMR spectroscopy of (**1**): 0 min., 24 h and 48 h, in DMSO (D_2O capillary).

Figure S9. ^{31}P˚^{1}H} NMR spectroscopy of (**1**): 0 min., 24 h and 48 h, in DMSO-DMEM mixture (D_2O capillary).

Figure S10. UV-vis spectra of (**2**): 0 min., 24 h and 48 h, in DMSO.

Figure S11. UV-vis spectra of (**2**): 0 min., 24 h and 48 h, in DMSO-DMEM mixture.

Figure S12. Experimental transitions (λ_{exp}, in nm) to each band detected in the HQ UV-Vis spectrum, along with computed PBE0 excitation energies (λ_{calc}, in nm) and associated oscillator strength (*F*, in a.u.), are provided. Additionally, the contribution from the charge transfer process (in %) to the calculated electronic transition, involving occupied and unoccupied orbitals, is included. An isovalue of 0.03 a.u. was applied to the electronic density of the orbitals.

Table S1. The mean absolute error (MAE) and the mean unsigned error (MUE) between the experimental and calculated values, involving bond lengths (in Å), angles (in degrees), and dihedral angles (in degrees), were assessed using different DFT methods employing the same basis sets.

Table S2. Experimental electronic transition (λ_{exp}, in nm) and computed PBE0 excitation energies (λ_{calc}, in nm) with associated oscillator strength (*F*, in a.u.) of (**1**).

Supporting Information

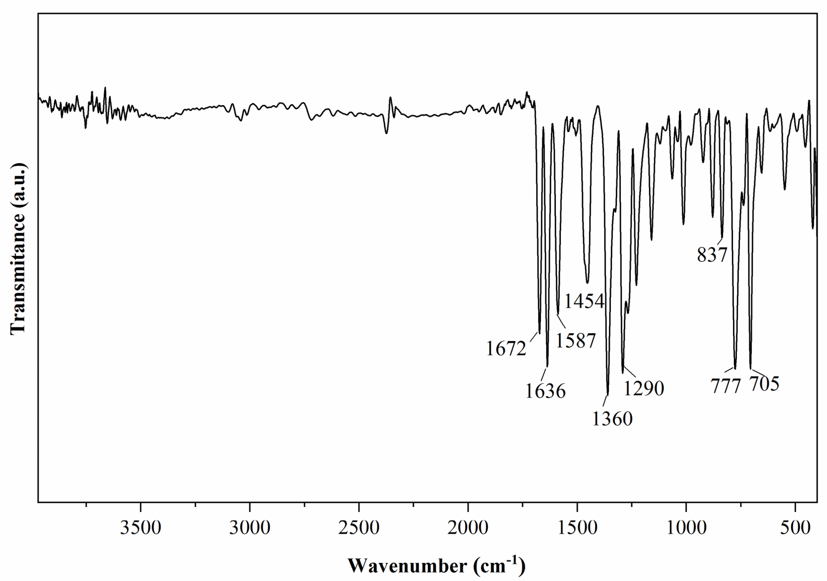

Figure S1. Infrared spectrum for HQ, in KBr pellet.

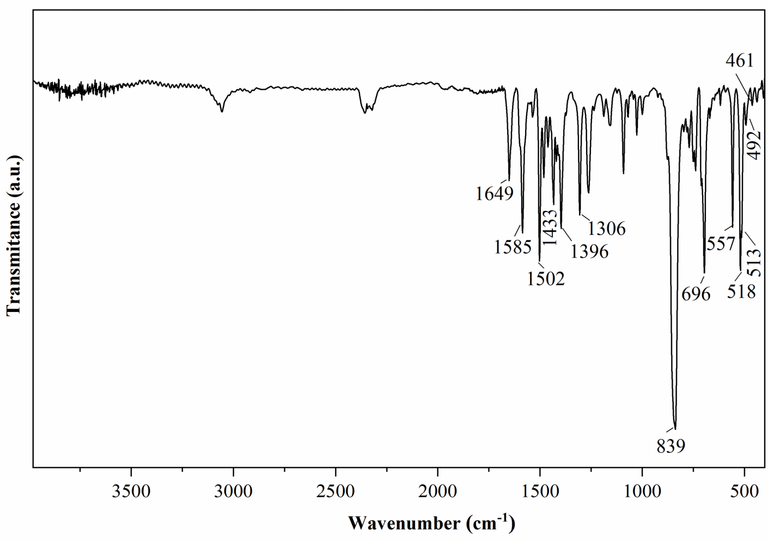

Figure S2. Infrared spectrum for (**1**), in KBr pellet.

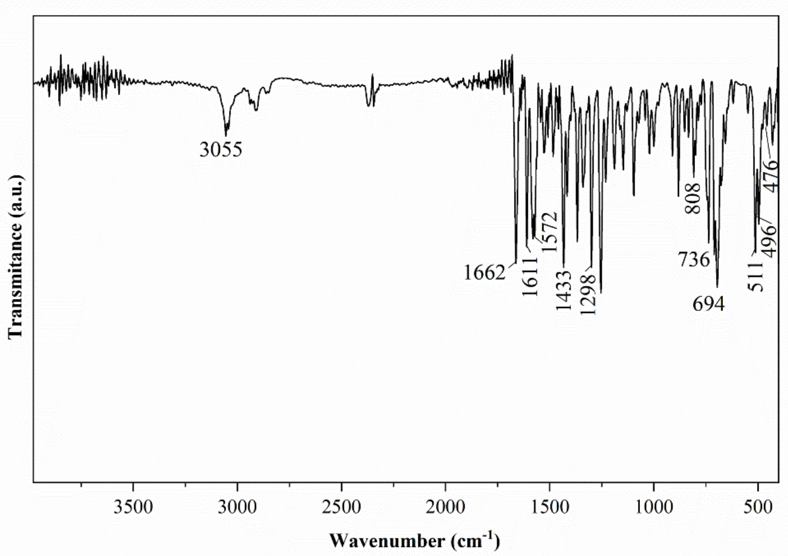

Figure S3. Infrared spectrum for (**2**), in KBr pellet.

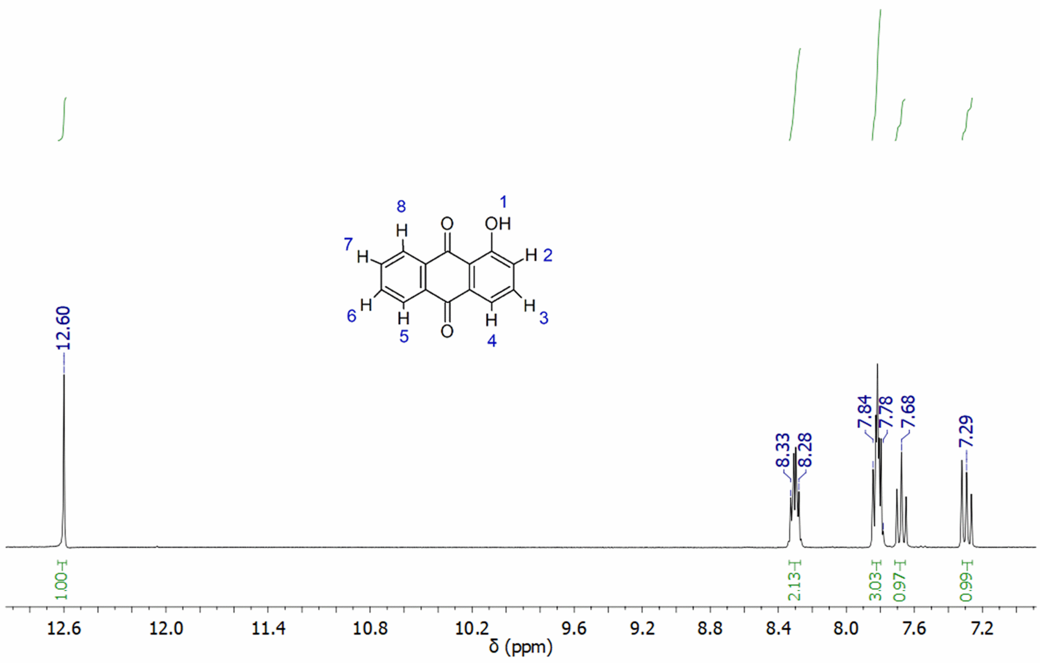

Figure S4. ^1H NMR spectrum for HQ, in $CDCl_3$.

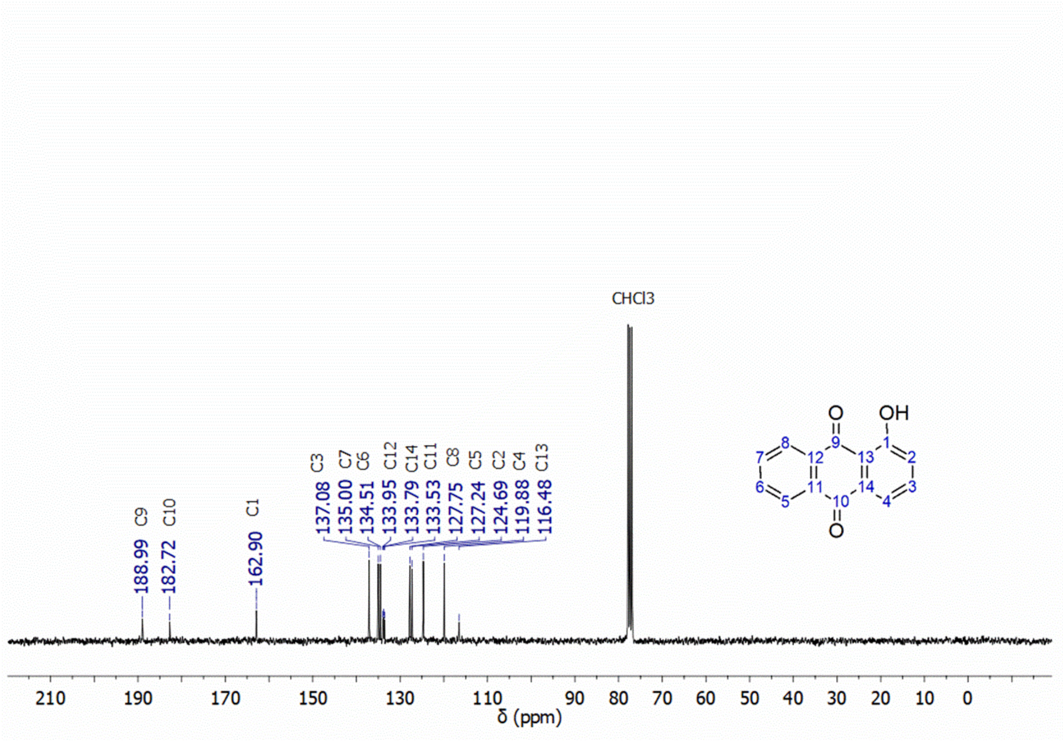

Figure S5. ^{13}C˚^{1}H} NMR spectrum for HQ, in CDCl$_3$.

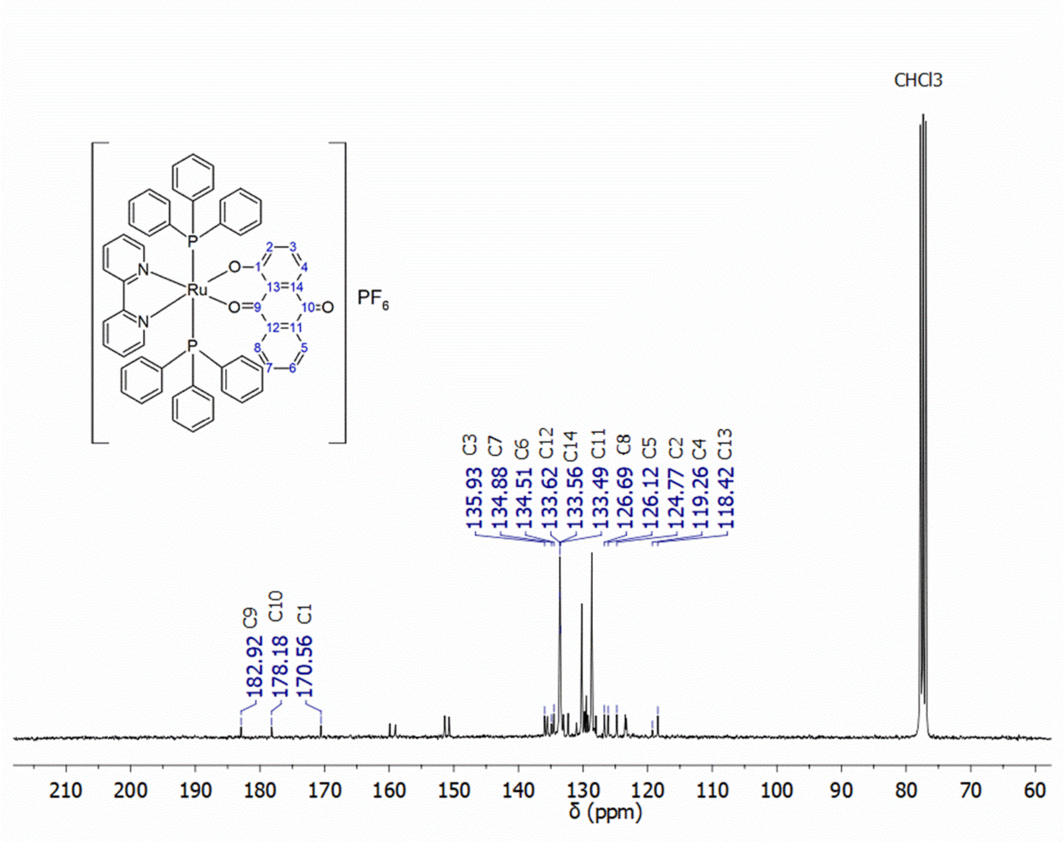

Figure S6. ^{13}C˚^{1}H} NMR spectrum for (**1**), in CDCl$_3$.

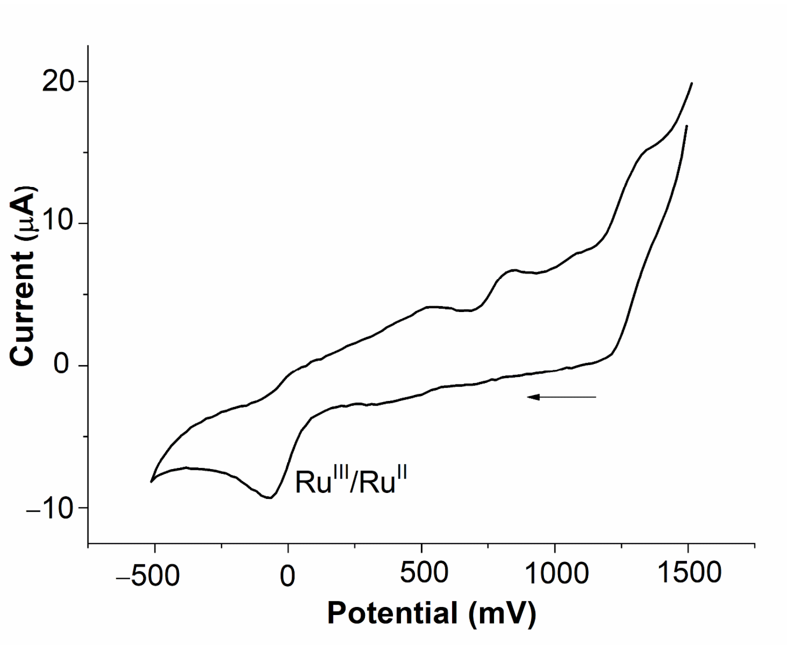

Figure S7. Cyclic voltammogram of (**P2**) (TBAP 0.1 M; CH_2Cl_2; Ag/AgCl; work electrode Pt; 100 mV s^{-1}).

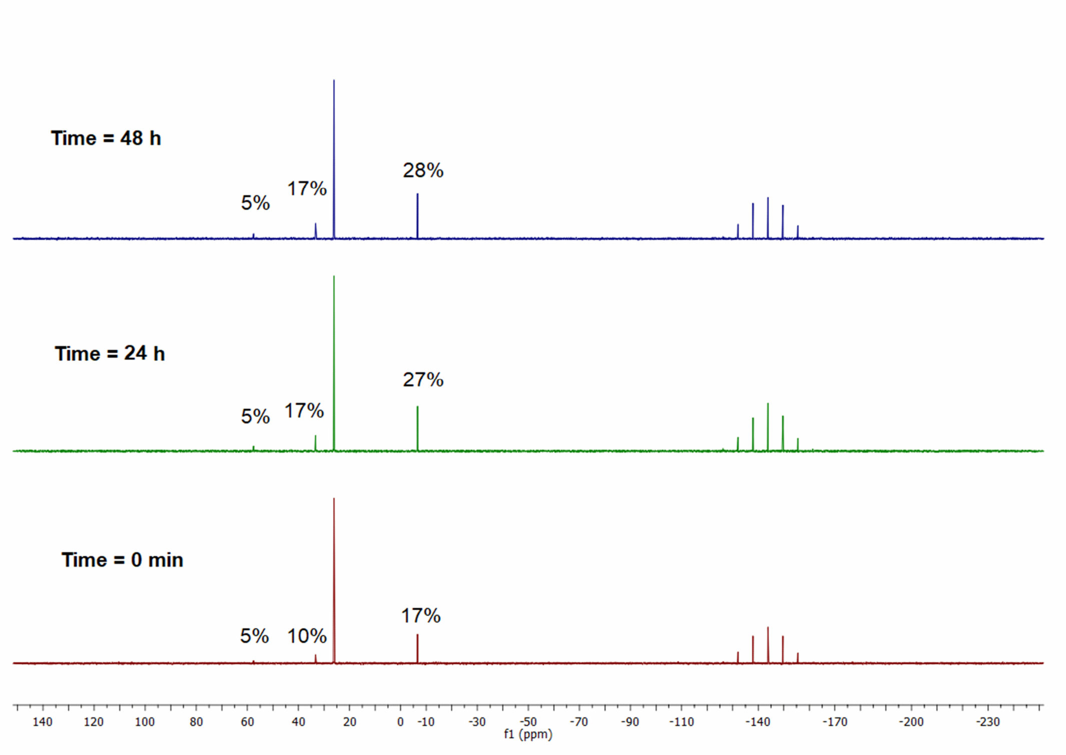

Figure S8. ^{31}P$^{\circ 1}$H} NMR spectroscopy of (**1**): 0 min, 24 h and 48 h, in DMSO (D_2O capillary).

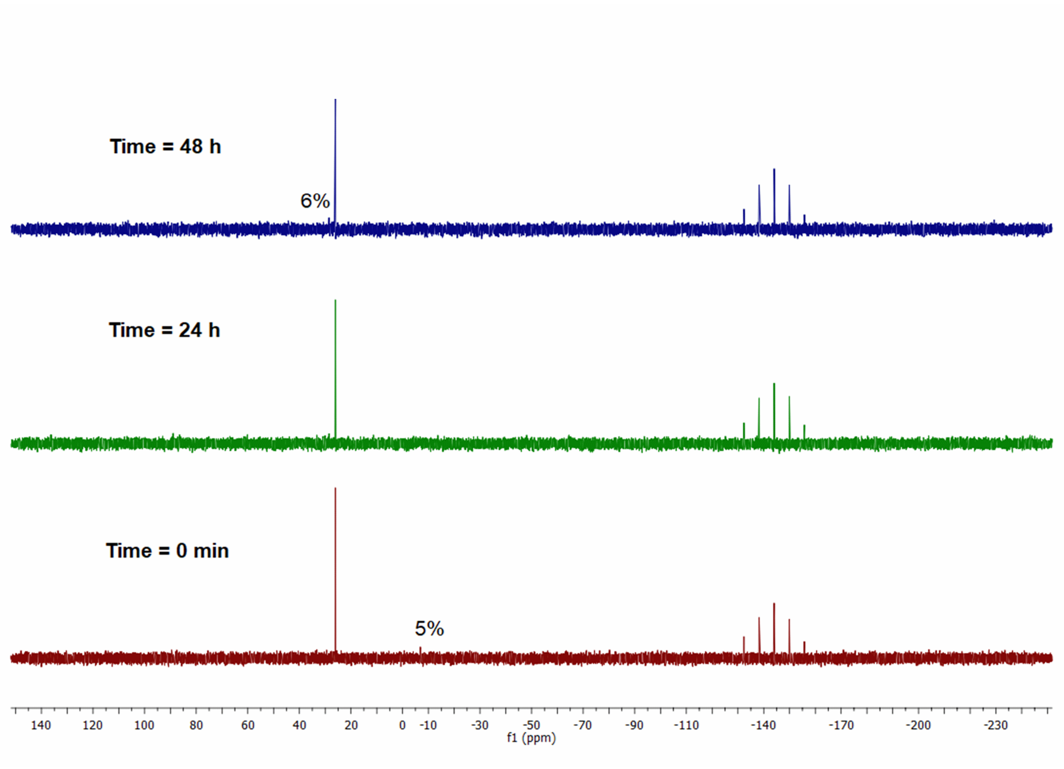

Figure S9. $^{31}P\{^{1}H\}$ NMR spectroscopy of (**1**): 0 min, 24 h and 48 h, in DMSO-DMEM mixture (D_2O capillary).

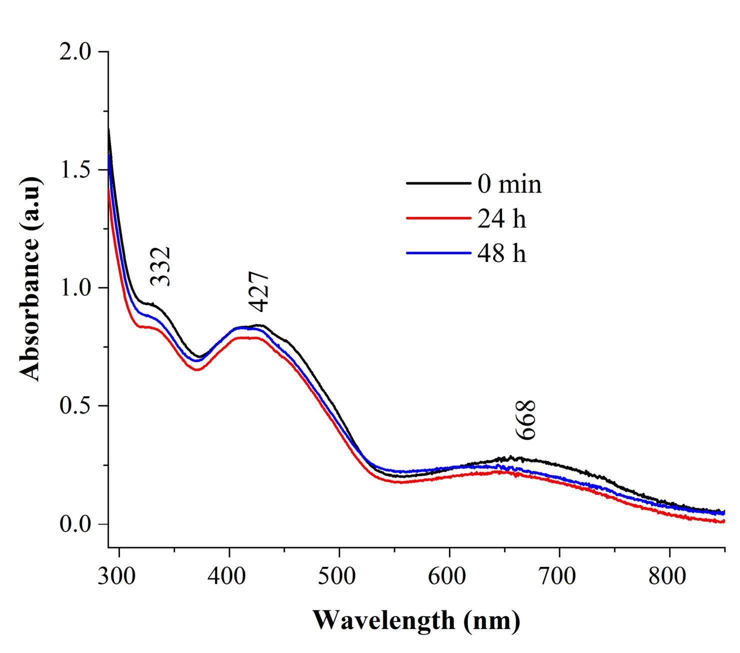

Figure S10. UV-vis spectra of (**2**): 0 min, 24 h and 48 h, in DMSO.

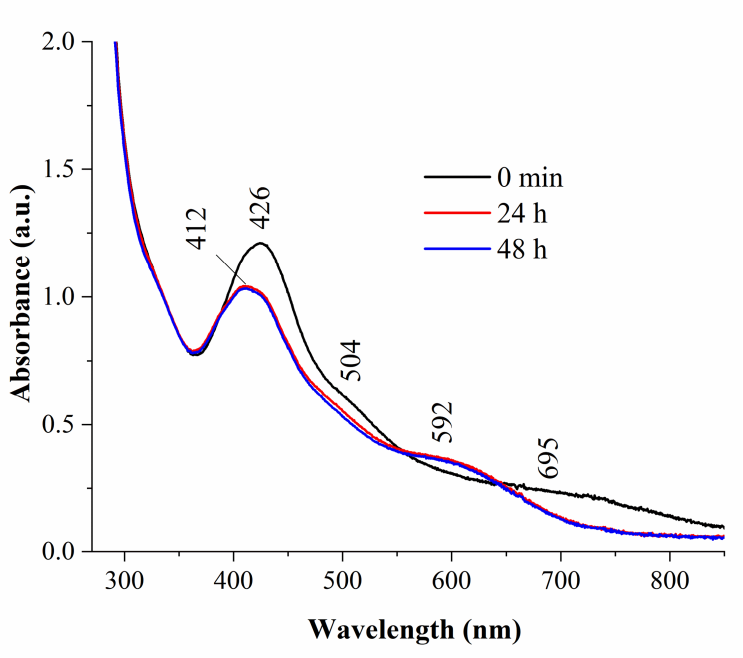

Figure S11. UV-vis spectra of (**2**): 0 min, 24 h and 48 h, in DMSO-DMEM mixture.

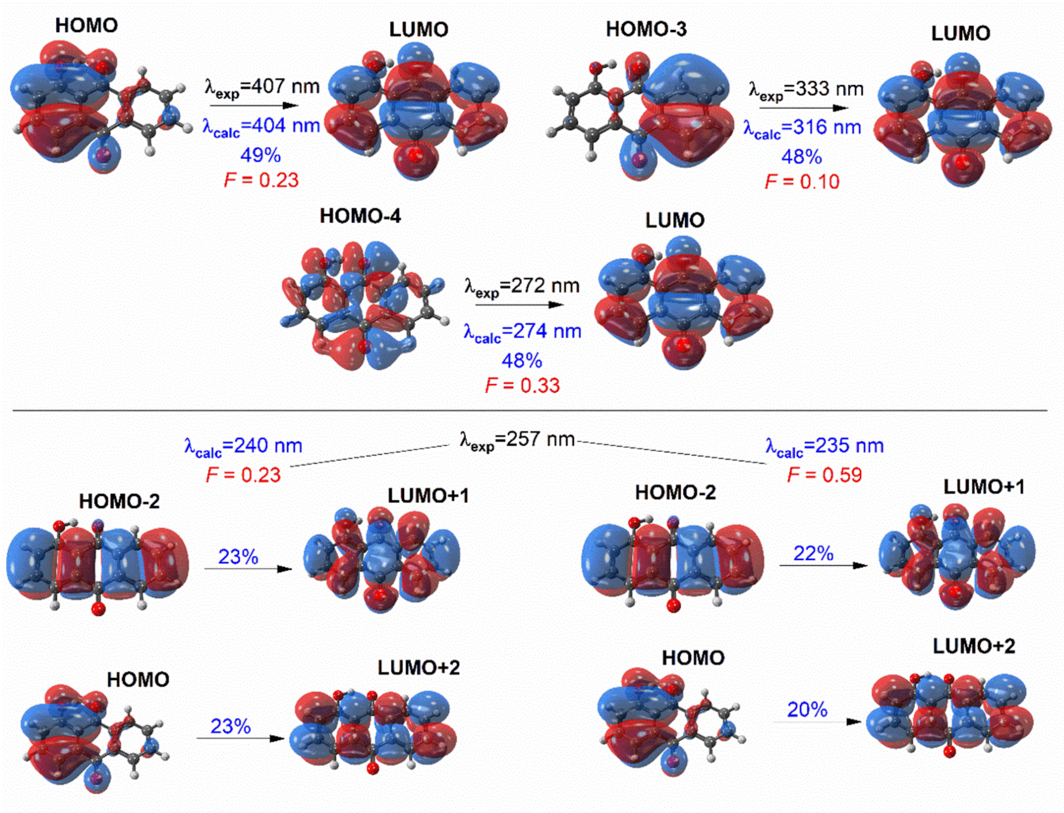

Figure S12. Experimental transitions (λ_{exp}, in nm) to each band detected in the HQ UV-Vis spectrum, along with computed PBE0 excitation energies (λ_{calc}, in nm) and associated oscillator strength (F, in a.u.), are provided. Additionally, the contribution from the

charge transfer process (in %) to the calculated electronic transition, involving occupied and unoccupied orbitals, is included. An isovalue of 0.03 a.u. was applied to the electronic density of the orbitals.

Table S1. The mean absolute error (MAE) and the mean unsigned error (MUE) between the experimental and calculated values, involving bond lengths (in Å), angles (in degrees), and dihedral angles (in degrees), were assessed using different DFT methods employing the same basis sets.

		PBE1PBE	M06L	M06	B3LYP	B97D
Bond lengths	MUE	0.069	0.070	0.070	0.074	0.078
	MAE	0.065	0.065	0.066	0.062	0.062
Angles	MUE	0.017	0.015	0.030	0.047	0.035
	MAE	0.79	0.74	0.88	1.08	0.77
Dihedral angles	MUE	-0.93	-5.01	-3.40	1.46	0.59
	MAE	60.35	65.71	63.86	58.35	58.06

Table S2. Experimental electronic transition (λ_{exp}, in nm) and computed PBE0 excitation energies (λ_{calc}, in nm) with associated oscillator strength (*F*, in a.u.) of (**1**).

			**Assignement**	**Character**
λ_{exp}	λ_{calc}	*F*	HQ	
407	403.8	0.23	π_{+}O-p $\rightarrow$ π^{*}_{+}O-p^{*} (49%)	
333	315.8	0.10	π_{+}O-p $\rightarrow$ π^{*}_{+}O-p^{*} (48%)	
272	274.3	0.33	σ-CC $_{+}$$lp$-O $\rightarrow$ π^{*}_{+}O-p^{*} (48%)	
257	239.8	0.23	π_{+}O-p $\rightarrow$ π^{*} (23%)<br>π $\rightarrow$ π^{*}_{+}O-p^{*} (23%)	
	234.6	0.59	π_{+}O-p $\rightarrow$ π^{*} (23%) //<br>π $\rightarrow$ π^{*}_{+}O-p^{*} (23%)	
λ_{exp}	λ_{calc}	*F*	(1)	
528	485.3	0.38	Ru-d $\rightarrow$ π^{*}_{HQ} (28%)<br>Ru-d + π_{HQ} $\rightarrow$ Ru-d^{*} + π^{*}_{bipy} (15%)	MLCT<br>MLMLCT
425	410.7	0.07	Ru-d $\rightarrow$ π^{*}_{HQ} (46%)	MLCT
350	367.8	0.09	Ru-d $\rightarrow$ π^{*}_{HQ} (43%)	MLCT
	353.7	0.04	Ru-d + π_{HQ} $\rightarrow$ Ru-d^{*} + π^{*}_{bipy} (30%)	MLMLCT
296	297.8	0.03	Ru-d $\rightarrow$ π^{*}_{PPh3} (37%)	MLCT
	296.5	0.03	Ru-d $\rightarrow$ Ru-d^{*} + π^{*}_{HQ} (18%)<br>Ru-d + π_{HQ} $\rightarrow$ π^{*}_{PPh3} (11%)	MMLCT<br>MLLCT
λ_{exp}	λ_{calc}	*F*	(2)	
668	639.8	0.01	Ru-d + Cl-p + π_{HQ} $\rightarrow$ π^{*}_{HQ} (59%)	MLLCT
	578.6	0.08	Ru-d + Cl-p + π_{HQ} $\rightarrow$ Ru-d^{*} + Cl-p^{*} + π^{*}_{HQ} (59%)	MLMLCT
455	501.7	0.03	Ru-d + Cl-p $\rightarrow$ Ru-d^{*} + π^{*}_{HQ} (42%)	MLMLCT

	472.8	0.05	Ru-*d* + Cl-*p* + π_{HQ} ⟶ π^*_{HQ} (15%)<br>Cl-*p* ⟶ Ru-d^* + Cl-p^* + π^*_{HQ} (16%)<br>Ru-*d* + Cl-*p* + π_{HQ} ⟶ Ru-d^* + π^*_{HQ} (18%)	MLLCT<br>LMLCT<br>MLMLCT
	449.3	0.04	Ru-*d* + Cl-*p* ⟶ π^*_{HQ} (14%)	MLLCT
	436.9	0.05	Cl-*p* + π_{HQ} + π_{dppb} ⟶ π^*_{HQ} (18%)<br>Ru-*d* + Cl-*p* ⟶ π^*_{HQ} (25%)	LLCT<br>MLLCT
341	388.2	0.02	Ru-*d* + Cl-*p* + π_{HQ} ⟶ π^*_{HQ} (48%)	MLLCT
286	352.8	0.02	Ru-*d* + Cl-*p* + π_{HQ} ⟶ π^*_{HQ} (14%)<br>Cl-*p* + π_{HQ} + π_{dppb} ⟶ Ru-d^* + Cl-p^* + π^*_{HQ} (16%)<br>π_{HQ} + π_{dppb} ⟶ Ru-d^* + Cl-p^* + π^*_{HQ} (18%)	MLLCT<br>LMLCT<br>LMLCT
	350	0.02	Cl-*p* + π_{dppb} ⟶ π^*_{HQ} (21%)	LLCT
**λ_{exp}**	**λ_{calc}**	***F***	**(P1)**	
488	454.8	0.08	Ru-*d* + Cl-*p* ⟶ Ru-d^* + π^*_{bipy} (46%)	MLMLCT
350	333.0	0.07	Ru-*d* + Cl-*p* ⟶ Ru-d^* + π^*_{bipy} (27%)	MLMLCT
	323.5	0.02	Ru-*d* + Cl-*p* ⟶ Ru-d^* + π^*_{bipy} (13%)<br>Ru-*d* + Cl-*p* ⟶ π^*_{PPh3} (30%)	MLMLCT<br>MLLCT
308	296.0	0.03	Ru-*d* + Cl-*p* + π_{PPh3}⟶ Ru-d^* + π^*_{bipy} (27%)<br>Ru-*d* + Cl-*p* ⟶ π^*_{PPh3} (16%)	MLMLCT<br>MLLCT
**λ_{exp}**	**λ_{calc}**	***F***	**(P2)**	
538	505.4	0.03	Ru-*d* + Cl-*p* ⟶ Ru-d^* + Cl-p^* (78%)	MLMLCT
416	465.2	0.01	Ru-*d* + Cl-*p* + π_{dppb} ⟶ Ru-d^* + Cl-p^* (32%)<br>Ru-*d* + Cl-*p* ⟶ Ru-d^* + Cl-p^* + π^*_{dppb} (17%)	MLMLCT<br>MLMLCT
	461.5	0.01	Ru-*d* + Cl-*p* ⟶ Ru-d^* + Cl-p^* (17%)<br>Ru-*d* + Cl-*p* + π_{dppb} ⟶ Ru-d^* + Cl-p^* (28%)<br>Ru-*d* + Cl-*p* ⟶ Ru-d^* + Cl-p^* + π^*_{dppb} (18%)	MLMLCT<br>MLMLCT<br>MLMLCT
	388.6	0.01	Ru-*d* + Cl-*p* + π_{dppb} ⟶ Ru-d^* + Cl-p^* (56%)	MLMLCT
355	354.1	0.01	Ru-*d* + Cl-*p* + π_{dppb} ⟶ Ru-d^* + Cl-p^* + π^*_{dppb} (16%)<br>Ru-*d* + Cl-*p* ⟶ Ru-d^* + Cl-p^* + π^*_{dppb} (17%)	MLMLCT<br>MLMLCT
	304.6	0.02	Ru-*d* + Cl-*p* ⟶ Ru-d^* + Cl-p^* (63%)	MLMLCT
260	275.4	0.01	Ru-*d* + Cl-*p* + π_{dppb} ⟶ Ru-d^* + Cl-p^* (10%)<br>Ru-*d* + Cl-*p* ⟶ Ru-d^* + Cl-p^* + π^*_{dppb} (6%)	MLMLCT<br>MLMLCT
